# Conserved helical motifs in the IKZF1 disordered region mediate NuRD interaction and transcriptional repression

**DOI:** 10.1182/blood.2024024787

**Published:** 2025-01-23

**Authors:** Tianyi Zhang, Yi-Fang Wang, Alex Montoya, Ilinca Patrascan, Nehir Nebioglu, Husayn A. Pallikonda, Radina Georgieva, James WD King, Holger B. Kramer, Pavel V. Shliaha, David S. Rueda, Matthias Merkenschlager

**Affiliations:** 1 MRC Laboratory of Medical Sciences, Institute of Clinical Sciences, Faculty of Medicine, Imperial College London, Du Cane Road W12 0HS; 2 Section of Virology, Department of Infectious Disease, Imperial College London, Du Cane Road, London W12 0HS

## Abstract

The transcription factor IKZF1 is essential for B cell development, and recurrently mutated in human B-ALL. IKZF1 has been ascribed both activating and repressive functions via interactions with coactivator and corepressor complexes, but the relative abundance of IKZF1-associated coregulators and their contribution to IKZF1-mediated gene regulation are not well understood. To address this, we performed an unbiased identification of IKZF1-interacting proteins in pre-B cells and found that IKZF1 interacts overwhelmingly with corepressors and heterochromatin-associated proteins. Time-resolved analysis of transcription and chromatin state identified transcriptional repression as the immediate response to IKZF1 induction. Transcriptional repression preceded transcriptional activation by several hours, manifesting as a decrease in the fraction of transcriptional bursts at the single molecule level. Repression was accompanied by a rapid loss of chromatin accessibility and reduced levels of H3K27ac particularly at enhancers. We identified highly conserved helical motifs within the intrinsically disordered region in IKZF1 that mediate its association with the NuRD corepressor complex through critical “KRK” residues that bind the NuRD subunit RBBP4, a mechanism shared with the TFs FOG1, BCL11A, and SALL4. Functional characterization reveals this region is necessary for to the efficient silencing of target genes and antiproliferative functions of IKZF1 in B-ALL.

## Introduction

In multicellular organisms, lineage-specific transcription factors (TFs) interact with coregulators to alter the chromatin state of gene regulatory elements, and promote or suppress transcription to orchestrate gene expression programs that drive differentiation^1^. IKZF1 is a hematopoietic TF essential for lymphoid commitment and pre-B cell development^2–4^. *IKZF1* haploinsufficiency and dominant negative mutations are frequent in pre-B cell acute lymphoblastic leukemia (B-ALL), where they predict poor patient outcomes^5–8^.

IKZF1 has been ascribed activating, repressive, pioneering, and 3D genome organizing functions^9–13^. Transcriptional control by IKZF1 has been linked to both the loss and gain of chromatin accessibility and epigenetic modifications at gene regulatory elements^9,12,14–16^. IKZF1 has been linked to corepressors^17^ including NuRD (Ref.^18^), Sin3 (Ref.^19^), HDACs, and CtBP1 (Ref.^20^). Repressive histone modifications H3K9me3 and H3K27me3 are thought to contribute to IKZF1-mediated transcriptional repression^15,21^. Conversely, a role for IKZF1 in gene activation is suggested by IKZF1 interactions with the BRG1/BAF chromatin remodeling complex^22–24^, the positive elongation factor P-TEFb (Ref.^25,26^), and the TFs Gata-1 (Ref.^25^) and Gfi1 (Ref.^14^). However, the relative abundance of coregulators associated with IKZF1, and their contribution to IKZF1-mediated gene regulation remain incompletely characterized, and is unclear precisely how IKZF1 binding modulates the chromatin environment to activate or repress transcription.

We find that the IKZF1 interactome is strongly biased towards corepressors over coactivators, and that transcriptional repression is the immediate response to IKZF1 induction in pre-B cells. IKZF1 controls target gene expression primarily through modulating the chromatin state of regulatory elements, in particular enhancers, which are rapidly attenuated by IKZF1. Regulatory elements most sensitive to IKZF1 repression have especially high IKZF1 motif density and are enriched for IKZF1 binding, recruitment of the NuRD complex, and rapid H3K27ac loss. Gene activation occurs later than repression, is generally not associated with direct IKZF1 binding, and therefore likely indirect. We identify and characterize conserved helices and a charged motif in the IKZF1 disordered region, which mediates IKZF1 association with NuRD, contributes to the stable silencing of target genes, and attenuates the proliferation of *IKZF1*-mutant human B-ALL cells.

## Methods

### Chromatin-associated RNA-seq

Adapted from^27^. Nuclei were isolated then lysed in Urea Buffer and chromatin-associated RNA was purified with Zymo Direct-zol RNA Miniprep. Libraries were made using NEBNext® Ultra™ II Directional RNA Library Prep with rRNA depletion.

### ATAC-seq

50K cells were lysed, and nuclei tagmented with 3ul of Ilumina Tn5 for 30min at 37°C. DNA was purified with Zymo DNA Clean and Concentrator and libraries were generated following the original ATAC protocol^28^.

### ChIP-seq

Adapted from ^29,30^. Cells were fixed with 1mM DSG and 1% formaldehyde. Nuclei were lysed and chromatin was digested to 100-400bp. IKZF1-ERt2 ChIP was performed with anti-HA beads (Pierce 88836). Samples were incubated overnight with 5μg of antibody (H3K27ac Active Motif 39133, CHD4 Abcam ab70469, H3K9me3 Diagenode C15410193, and KAP1 Abcam ab10483), 2h with ProtA/G Dynabeads, washed with Low-Salt, High-Salt, LiCl Buffer, and TE. Eluate was reverse-crosslinked at 65°C with 0.2M NaCl, 200μg/ml RNaseA, and 200μg/ml Proteinase K, then purified with Zymo ChIP DNA Clean and Concentrator. Libraries were made with NEBNext® Ultra™ II DNA Library Prep.

### Next-generation sequencing

75bp paired-end for RNA and ATAC, and 40bp paired-end for ChIP were perfomed on the Illumina NextSeq500.

### ChIP MS

Adapted from ^31^. Cells were fixed in 1% formaldehyde, and chromatin was sonicated to 100-300bp. Pulldown was performed with anti-HA beads (Pierce 88836), and washed 3x in Low Salt, 3x High Salt, 2x in LiCl Buffer, 2x in 20 mM EPPS.

### Affinity-purification MS

Nuclear extracts obtained from salt extraction were incubated with anti-HA beads, washed 4x in Wash Buffer with then without 0.2% NP-40, then 2x in 20 mM EPPS. See supplemental methods for MS run parameters.

### Nascent RNA-FISH

B3 were seeded at 1million/well in 18-well slides (Ibidi, 81816), and intronic smRNA-FISH (Thermofisher probes) was performed with View RNA ISH cell assay kit. Burst fraction was calculated as the number of bursts divided by the number of alleles (2 x total cells). Burst size was the sum of Intensity_MaxIntensity and AreaShape_MeanRadius from CellProfiler v4.2.1.

### Cell line generation

B3 cells with IKZF1 constructs were made using retroviral plasmids MSCV-IKZF1-IRES-GFP and pCL-Eco. IKZF1 B-ALL cells were made with lentiviral plasmids pLVX-IKZF1-ERt2-IRES-ZsGreen, psPAX2, pMD2.G. Cells were GFP-sorted 3d post-infection. 500nM of 4-hydroxytamoxifen was used for IKZF1-ERt2 induction.

### Cell growth assay

B-ALL were seeded at 2x10^5^ cells/ml in RPMI, 10% FBS, Pen/Strep, L-glutamine, and counted every other day.

### Cell Cycle Analysis

500K cells were fixed in 0.5ml ice-cold 70% ethanol, and stained in 250ul Propidium iodide (PI) in PBS for 30 min on ice. 40-50K events for each sample were recorded using LSRFortessa (BD Biosciences) and analysed using the Cell Cycle tool in FlowJo v10.

### GSEA

Gene set enrichment^32^ was performed for early IKZF1-induced DE genes (2h vs. 0h) against gene sets Reactome integrin cell surface interaction, Reactome cell cycle, down or upregulated in DN Ik6 B-ALL vs wildtype IKZF1 B-ALL (DE padj<0.01 and FC>1.5 from Supplementary Table 4 (Ref.^33^).

### Homer motifs

Homer findMotifsGenome.pl was performed on ATAC NFR peaks with early-decreased or increased accessibility (2h vs 0h padj<0.01) as target, and unchanged-accessibility regions as background with “-size given” for size of regions and “-mm10r” for masked-genome.

### Motif count and density

Motif count was the number of IKZF1 motifs GGAA, TTCC, GGGA, TCCC per ATAC-seq peak, and density was calculated as count/(peak length in bp).

Supplementary methods include buffer compositions, qPCR primers, analysis pipelines.

## Results

### IKZF1 interactome is strongly biased towards corepressors over coactivators

**Figure 1:**
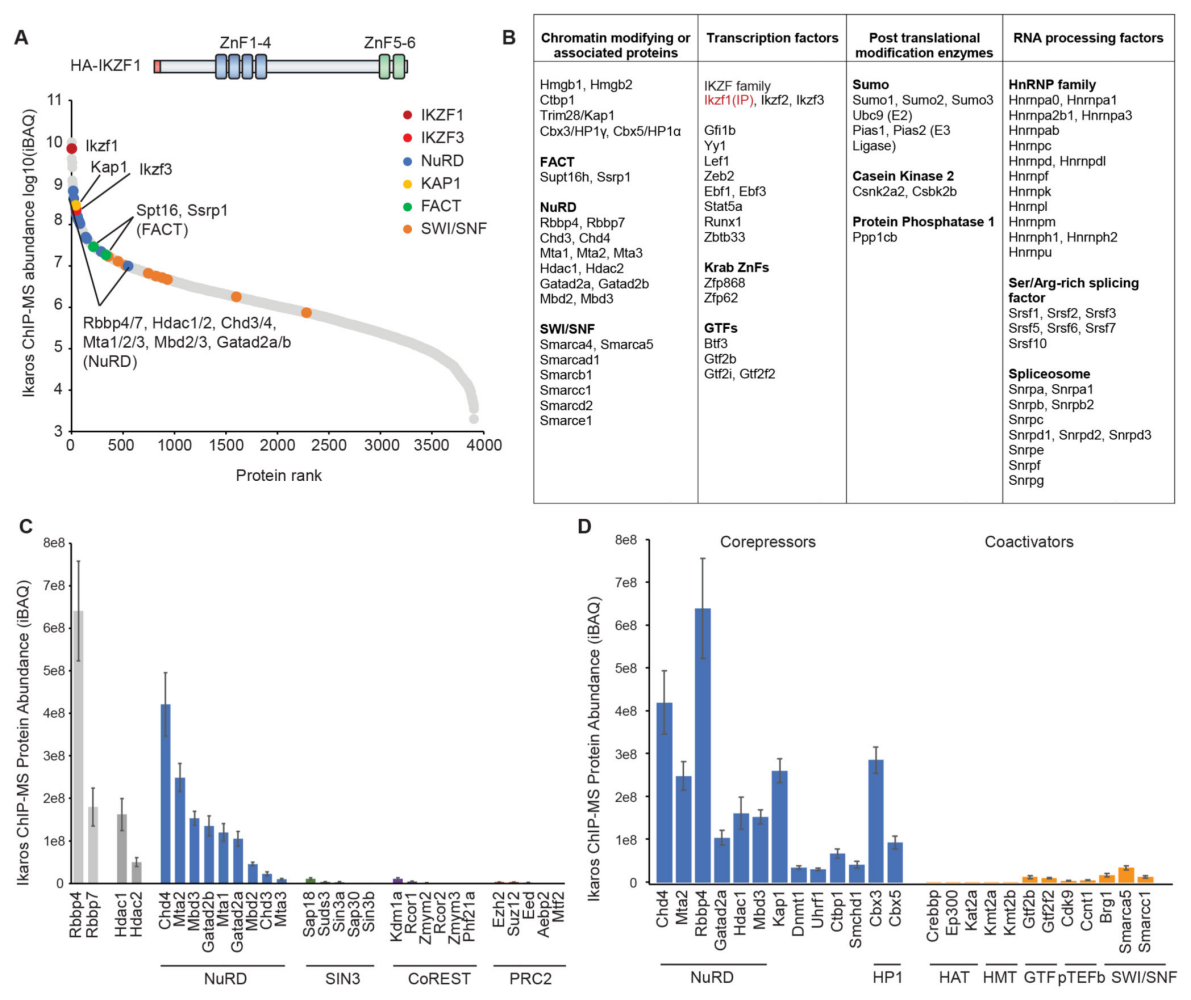
IKZF1 interactome is strongly biased for corepressors over coactivators A. IKZF1 ChIP-MS interacting proteins ranked by abundance (log10 iBAQ mean of three replicates), with IKZF1 in maroon, IKZF3 (Aiolos) in red, NuRD subunits in blue, KAP1 in yellow, FACT subunits in green, and SWI/SNF subunits in orange. B. Top 20% most abundant IKZF1 ChIP-MS interacting proteins, classified into chromatin coregulators, transcription factors, post-translational modification enzymes, and RNA-processing factors. C. iBAQ abundance of HDAC1/2 and RBBP4/7-containing complexes NuRD, SIN3, and CoREST, and the RBBP4/7-containing complex PRC2. Average and standard deviation of three replicates. D. Most abundant IKZF1 ChIP-MS corepressors versus coactivators. Average and standard deviation of three replicates.

To identify IKZF1-associated coregulators, we performed ChIP mass spectrometry of HA-tagged IKZF1 in B3 cells, which have a gene expression profile that closely resembles pre-B cell progenitors (Hardy’s Fr C’)^34^. We identified ∼4000 IKZF1 chromatin-mediated interactors (Fig. 1A, Supplementary Fig. 1A) with DNA-binding and chromatin-associated proteins among the most enriched. IKZF3, a family member that heterodimerizes with IKZF1 (Ref^35^), and B-cell TFs GFI1b, LEF1, ZEB2, EBF1, STAT5A, RUNX1 were highly abundant (Fig. 1B). Other top interactors included RNA-processing factors and enzymes involved in posttranslational modification of IKZF1 including CK2 kinase (Ref^36^), PP1 phosphatase Ref^37^ and PIAS sumo-ligases^38^ (Fig. 1B). IKZF1 affinity-purification MS from nuclear extracts without crosslinking yielded similar top interactors (Supplementary Fig. 1BC), indicative of direct protein-protein interactions.

Top interactors were associated mainly with repressive functions such as histone deacetylation, DNA methylation, and heterochromatin formation, including known IKZF1-associated corepressors CtBP1 (Ref^20^) and NuRD (Ref^18^, Fig. 1B). NuRD was the predominant IKZF1-associated HDAC complex, while HDACs SIN3 (Ref^19^) and CoREST were >40-fold less abundant (Fig. 1C). Novel IKZF1-interactors associated with heterochromatin formation, DNA methylation, and H3K9 methylation included KAP1, DNMT1, UHRF1, SMCHD1, and HP1 (Fig. 1BD). By contrast, facultative heterochromatin factors such as PRC2 were extremely low (Fig. 1C).

Hence, mass spectrometry of IKZF1 interactors showed that IKZF1 was significantly more highly associated with corepressors than with coactivators, both on chromatin (Fig. 1D) and in the nuclear soluble fraction (Supplementary Fig. 1BC). Previously reported interactors such as the transcription elongation factor P-TEFb (Ref^25^) and BRG1 SWI/SNF complex^22^ were >10-fold less abundant than NuRD (Fig. 1D). Chromatin-mediated interactions of IKZF1 with coactivators p300/CBP, H3K4 methyltransferases and general TFs were 20 to 2000-fold less abundant than NuRD (Fig. 1D). We conclude that in pre-B cells IKZF1 overwhelmingly associates with corepressors.

### Transcriptional repression is the immediate response to IKZF1 induction in pre-B cells

**Figure 2:**
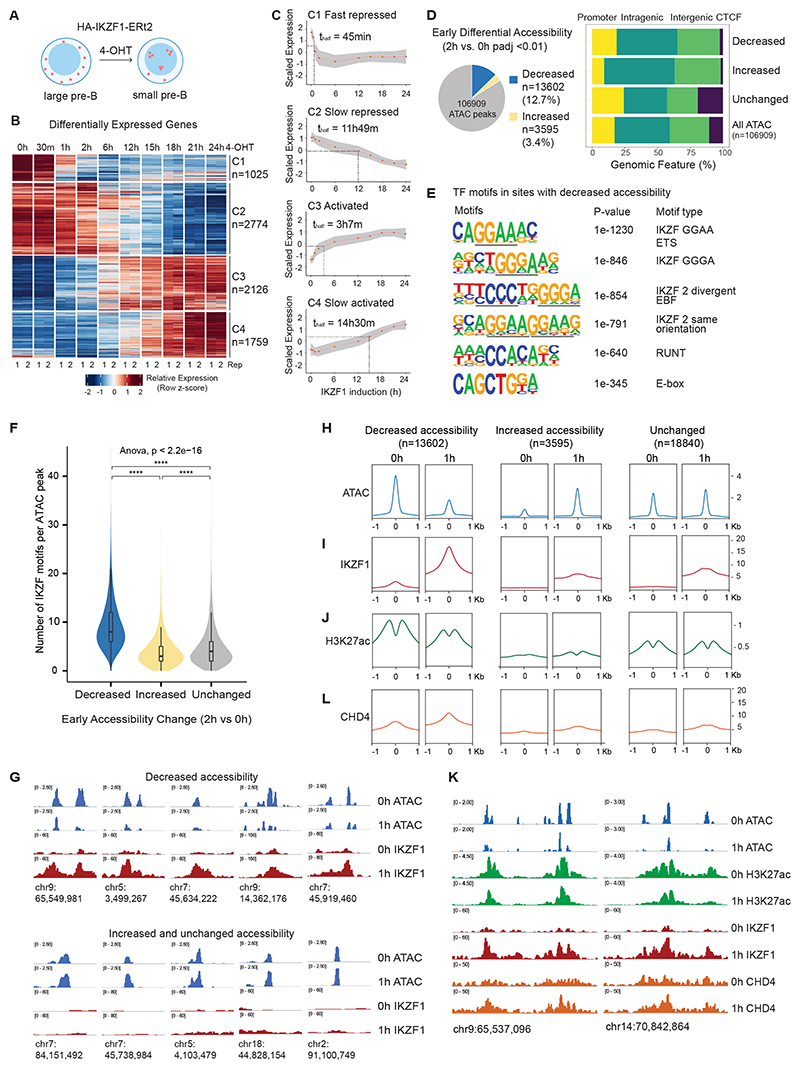
IKZF1 induces rapid loss of chromatin accessibility and H3K27ac at selective sites across the genome A. Inducible IKZF1-ERt2 pre-B model, where 4-Hydroxytamoxifen (4-OHT) induces synchronous nuclear entry of IKZF1-ERt2. B. Heatmap of differentially expressed genes (padj <0.01 and log2FC>1 or <-1) over 24h nascent chromatin-associated RNA-seq timecourse following IKZF1 induction performed in duplicate, and K-means clusters of genes by behaviour into C1 Fast Repressed, C2 Slow Repressed, C3 Activated and C4 Slow Activated. C. Scaled normalized expression for K-means clusters C1 Fast Repressed, C2 Slow Repressed, C3 Activated, and C4 Slow Activated genes, with red line and grey area indicating mean and 95% confidence intervals respectively. The time for genes in each cluster to reach half-maximal repression or activation is indicated by t-half. D. Left – Total Nucleosome Free Region (NFR) ATAC peaks called by MACS2 from ATAC-seq performed in duplicate and proportion of ATAC peaks with significant differential accessibility within 2h of IKZF1 induction, decreased accessibility in blue (2h vs 0h padj<0.01, FC<0), and increased accessibility in yellow (2h vs 0h padj <0.01, FC>0). Right - Relative genomic distribution of ATAC NFR peaks at CTCF sites, Promoters, Intragenic, and Intergenic for sites with early decreased accessibility, early increased accessibility, and a subset of sites with unchanged accessibility (padj>0.05, log2FC[-0.2,0.2]), and all accessible sites. E. Homer motif analysis showing top enriched motifs at ATAC NFR regions with early decreased accessibility compared to regions with unchanged accessibility. F. Number of IKZF motif (GGAA and GGGA) at sites with early decreased accessibility, increased accessibility, or unchanged accessibility. G. IGV browser track of ATAC and IKZF1-ERt2 at 0h and 1h following IKZF1 induction at sites with early decreased accessibility (top panel) and sites with early increased or unchanged accessibility (bottom panel) H. Metaprofile plot of chromatin accessibility at 0h and 1h following IKZF1 induction at +/-1kb around sites centered at ATAC peaks with early decreased, increased or unchanged accessibility. I. Metaprofile plot of IKZF1-ERt2 binding at 0h and 1h following IKZF1 induction at sites defined in H. J. Metaprofile plot of H3K27ac levels at 0h and 1h following IKZF1 induction at sites defined in H. K. IGV browser tracks of genomic regions that show early decreased accessibility, with ATAC in blue, H3K27ac in green, IKZF1-ERt2 in red, and CHD4 in orange before (0h) and 1h following IKZF1 induction. L. Metaprofile plot of CHD4 binding at 0h and 1h following IKZF1 induction at sites defined in H.

To address how IKZF1 and associated coregulators modify chromatin to regulate transcription, we employed an inducible IKZF1 model based on tamoxifen-induced nuclear translocation of IKZF1-ERt2 (Fig. 2A). The temporal resolution afforded by this approach identifies immediate effects of IKZF1 on chromate state and transcription.

Chromatin-associated RNA-seq, which enriches for nascent transcripts, detected transcriptional changes in 139 genes (126 down, 13 up) within 30 min of IKZF1 induction, and in 8165 genes over the entire 24h time-course (padj<0.01, FC>2) (Fig. 2B). We separated differentially-expressed genes by behavior using K-means clustering (Fig. 2B). Genes in C1 and C2 were repressed, and in C3 and C4 were activated (Fig. 2BC, Supplementary Table 1). A small cluster of non-monotonic genes (C5) enriched in sterol pathways linked to tamoxifen treatment was removed from further analysis^39,40^ (Supplementary Fig. 2AB). To determine the speed of transcriptional changes, we calculated the time taken for half-maximal repression or activation (Fig. 2C).

The most immediate response to IKZF1 induction was rapid repression of genes in C1. Half-maximal repression of these fast-repressed genes was reached within 45min and full repression within 2h (Fig. 2C). These fast-repressed genes were enriched in pathways related to immune processes, leukocyte differentiation, cell migration, and adhesion (Supplementary Fig. 2B), and included previously characterized IKZF1-targets such as *Igll1* (Ref.^41,42^), *VpreB1* (Ref.^43^) and *Dntt (*Ref.^44^). Transcriptional responses of C2, C3, and C4 genes were slower, suggesting they may not be direct targets of IKZF1 regulation. C2 slow-repressed genes were enriched for metabolic pathways, and reached half-maximal repression around 12h (Fig. 2C, Supplementary Fig. 2B). C3 activated and C4 slow-activated genes were enriched in immunoglobulin production and defense responses and half-maximally activated by 3h and 14.5h, respectively (Fig. 2C, Supplementary Fig. 2B). Hence, the immediate consequence of IKZF1 induction was transcriptional repression, which preceded transcriptional upregulation by 2.5h or more.

### IKZF1 binding drives rapid loss of chromatin accessibility and H3K27ac at promoters and enhancers

To understand how rapid transcriptional repression is accomplished, we monitored IKZF1 binding and changes to the chromatin landscape during the first 2h of induction. We used ATAC-seq and ChIP-seq for histone H3K27 acetylation to map changes in chromatin accessibility and activity of regulatory elements^45,46,47^. IKZF1-induction rapidly diminished chromatin accessibility at a subset of sites across the genome. Using ATAC-seq reads corresponding to nucleosome-free regions (NFR) which are enriched for TF binding^48^, we called 106909 ATAC peaks (Fig. 2D left). Within 2h of IKZF1 induction, 12.7% of sites significantly decreased in accessibility, while 3.4% increased in accessibility (padj<0.01, Fig. 2D left). Early differentially-accessible sites overlapped promoters, intragenic, and intergenic regions, but IKZF1-induction did not affect the accessibility of CTCF sites (Fig. 2D right).

Sites with early-decreased accessibility were highly enriched for IKZF1 binding-motifs (Fig. 2E), and contained a significantly higher number and density of IKZF1 motifs than sites with early-increased or unchanged accessibility (Fig. 2F, Supplementary Fig. 2C). Accordingly, IKZF1 ChIP-seq showed strong enrichment of IKZF1 binding at early-decreased accessibility sites, but not at sites with early-increased or unchanged accessibility (Fig. 2GHI). Notably, IKZF1 peak-summits were precisely centered at ATAC peaks with early-decreased accessibility, but not at sites with increased or unchanged accessibility (Fig. 2GHI). Sites with early-increased accessibility were enriched for other TF motifs including E-box, KLF, Forkhead, and Runt (Supplementary Fig. 2D), indicating that early-increased accessibility was independent of IKZF1 binding.

Most (68%) early-decreased accessibility sites overlapped H3K27ac, a signature of active promoters and enhancers (Supplementary Fig. 2E). These sites showed marked loss in H3K27ac upon IKZF1 binding (Fig. 2JK, Supplementary Fig. 2F). These data support a mechanism of repression where IKZF1 binding drives rapid loss of focal accessibility, followed by reduced H3K27ac across the broader region.

### Early NuRD recruitment to IKZF1-repressed sites

The speed and extent of decrease in chromatin accessibility and H3K27ac suggested a process of active chromatin remodeling and deacetylation. To explore the underlying mechanisms, we focused on NuRD and KAP1 as the most abundant corepressors identified by ChIP-MS. NuRD combines chromatin remodeling and histone deacetylase activity, while KAP1 promotes spreading of repressive H3K9me3 (Ref^49^). ChIP-seq of CHD4, the chromatin remodeling subunit of NuRD, showed increased CHD4 binding after IKZF1 induction that closely mirrored the binding of IKZF1 itself (Fig. 2IKL). As observed for IKZF1, CHD4 peaks were precisely centered and most enriched at sites with early-decreased accessibility (Fig. 2KL). Unlike IKZF1 and CHD4, which bound strongly at accessible sites, KAP1 and H3K9me3 peaks were depleted from accessible sites (Supplementary Fig. 2GH). KAP1 and H3K9me3 were not enriched at early-decreased accessibility sites following IKZF1 induction (Supplementary Fig. 2GH). Together, these data suggest that rapid loss of chromatin accessibility and H3K27ac from active regulatory elements in response to IKZF1 induction is associated with early NuRD recruitment, but not KAP1 or H3K9me3.

### Enhancers are highly sensitive to IKZF1-mediated repression

**Figure 3:**
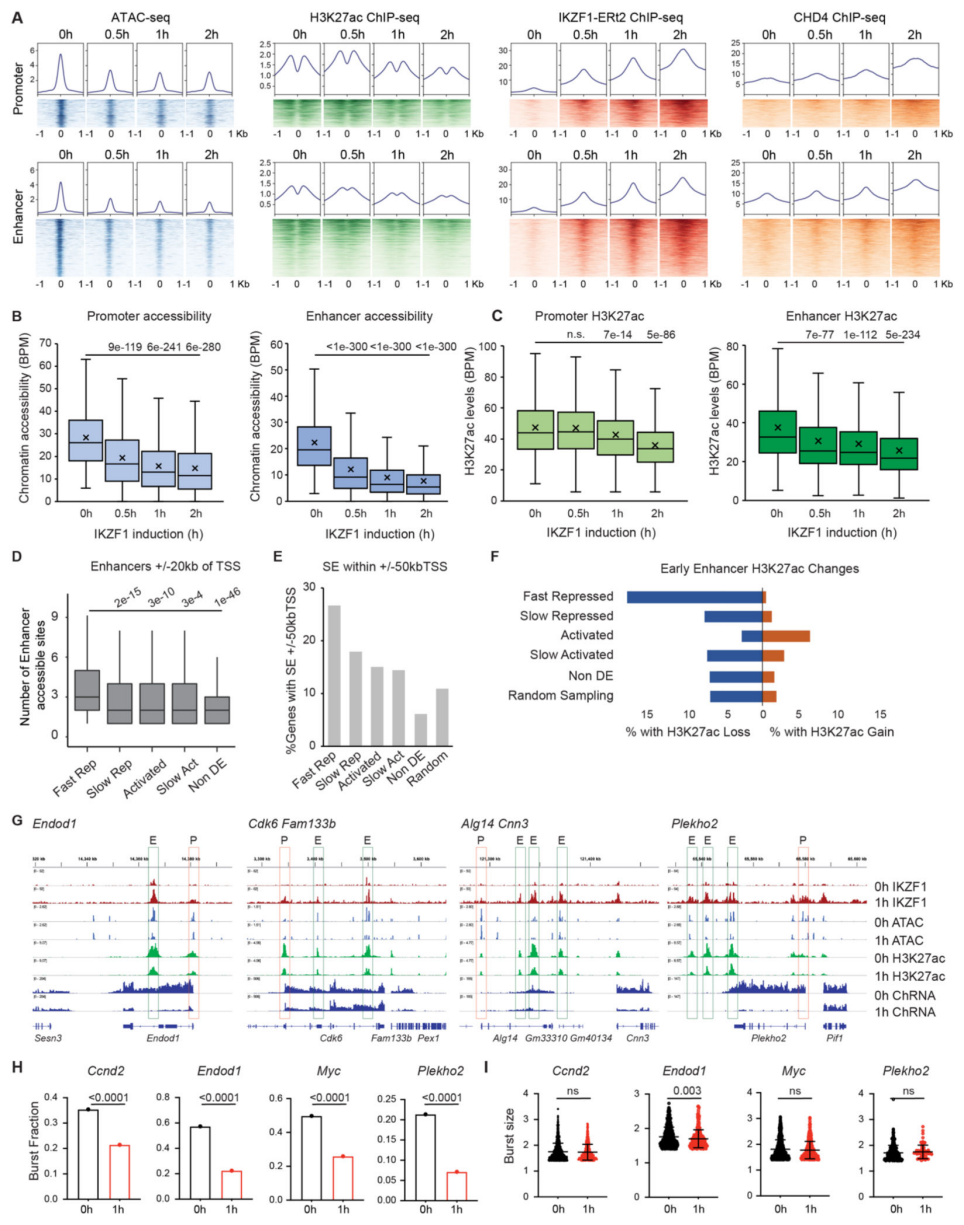
IKZF1 suppresses enhancer activity to repress transcription A. Heatmap and metaprofile plot of chromatin accessibility, H3K27ac, IKZF1-ERt2, and CHD4 at 0h, 30m, 1h, and 2h following IKZF1 induction +/-1kb of ATAC sites with early decreased accessibility overlapping active promoters (n=2420) or enhancers (n=6273). B. Boxplot of chromatin accessibility at active promoters and enhancers before (0h) and at early timepoints following IKZF1 induction (30m, 1h, 2h). Pairwise student’s t-test was performed for 0h vs 30m, 1h, or 2h with Bonferroni corrected p-adjusted value stated. C. Boxplot of H3K27ac at active promoters and enhancers before (0h) and at early timepoints following IKZF1 induction (30m, 1h, 2h). Pairwise student’s t-test was performed for 0h vs 30m, 1h, or 2h with Bonferroni corrected p-adjusted value stated. D. Number of accessible peaks within active enhancers in the region +/- 20kb TSS of Fast Repressed, Slow Repressed, Activated, Slow Activated, and non-differentially expressed genes. Pairwise Wilcoxon rank sum test was performed for Fast Repressed vs other gene class with Bonferroni corrected p-adjusted values stated. E. Proportion of genes that overlap with a SE within +/-50kb of the TSS, for Fast Repressed, Slow Repressed, Activated, Slow Activated, non-differentially expressed, and a randomly sampled set of genes. F. Proportion of enhancer H3K27ac peaks within +/- 20kb of TSS Fast Repressed, Slow Repressed, Activated, Slow Activated, non-differentially expressed, and randomly sampled genes that show significant (padj<0.01, FC>1.5) loss or gain of H3K27ac within 2h of IKZF1 induction. G. IGV browser of IKZF1 repressed genes at 0h and 1h after IKZF1 induction for ATAC in blue, H3K27ac in green, IKZF1-ERt2 in red, chRNA-seq and Refseq genes in navy, and boxes around promoters (P) and enhancers (E) peaks. H. Fraction of alleles with transcriptional bursting, detected using intron probes against IKZF1 repressed genes *Ccnd2, Endod1, Myc*, and *Plekho2* at 0h and 1h after IKZF1 induction. Approximately 700 cells (x2 alleles) were analyzed for each condition. P-value was calculated using the Fisher’s Exact Test. I. Transcriptional burst size of IKZF1 repressed genes *Ccnd2, Endod1, Myc*, and *Plekho2* at 0h and 1h after IKZF1 induction. P-value was calculated by unpaired Student’s t-test.

We next analyzed IKZF1-mediated repression of active promoters and enhancers. Genome-wide, active (H3K27ac-marked) enhancers were twice as likely to lose accessibility as promoters. Within 2h of IKZF1 induction, 30% of accessible sites within active enhancers showed significantly decreased accessibility, compared with 15% of accessible sites within active promoters (Supplementary Fig. 3A). While the levels of IKZF1 and CHD4 binding were similar at active enhancers and promoters with early-decreased accessibility, enhancers lost accessibility faster and to a greater extent than promoters (Fig. 3AB). Differences between promoters and enhancers were even more pronounced for the loss of H3K27ac (Fig. 3C). Significant H3K27ac loss at active enhancers occurred within 30min of IKZF1 induction, whereas H3K27ac at promoters did not decrease until 1h (Fig. 3C). Moreover, sites with the greatest reduction in H3K27ac more often overlapped enhancers than promoters (Supplementary Fig. 3B). Therefore, IKZF1 represses accessibility and H3K27ac at enhancers more rapidly and to a greater extent than promoters.

### Preferential association of fast-repressed genes with enhancers

Consistent with a model where enhancers are primary targets of IKZF1-mediated repression, fast-repressed genes were associated with a higher local density of enhancers (+/-20kb, Fig. 3D) and these enhancers had a greater number of IKZF1-bound sites (Supplementary Fig. 3C). A greater proportion of fast-repressed genes (26%) were associated with super-enhancers (SEs) compared to slow-response and non-differentially-expressed genes (Fig. 3E). To assign enhancers to promoters by a data-driven approach, we used Fantom5 CAGE-seq to correlate enhancer and gene transcription and Hi-C contact frequency (Supplemental Methods). This analysis established that fast-repressed genes had a greater number of enhancer associations (Supplementary Fig. 3D).

Not only were fast-repressed genes associated with more enhancers, these enhancers also exhibited a greater loss in accessibility and H3K27ac compared to enhancers associated with slow-response genes (Fig. 3F, Supplementary Fig. 3E). Enhancers near fast-repressed genes were approximately twice as likely to lose accessibility and three times more likely to lose H3K27ac compared with enhancers near randomly-sampled genes (Supplementary Fig. 3FG). By comparison, enhancers associated with other classes were less likely to lose accessibility and H3K27ac (Fig. 3F, Supplementary Fig. 3FG). We observed numerous examples of IKZF1-bound enhancers that were associated with fast-repressed genes, bound by IKZF1 binding, and rapidly lost accessibility and H3K27ac (Fig. 3G). Fast-repressed genes also clustered within the same topologically associated domains (Supplementary Figure 3H) more often than expected (Supplementary Fig. 3IJ). IKZF1 had a pronounced impact on SEs: almost half of all SEs (45%) that lost H3K27ac were associated with fast-repressed genes (i.e. *Bcl11a, Ccnd2, Endod1, Igll1, Itga5*, and *Gfra2*) (Supplementary Fig. 3KL). Taken together, these results suggest that enhancer disruption may be a primary driver of IKZF1-mediated transcriptional repression.

### IKZF1 disrupts the probability of transcriptional bursting of target genes

Transcription is a discontinuous process occurring in bursts^50,51^, and can be regulated by the probability (frequency) or the intensity (size) of bursting^50,51^. While these parameters have been extensively characterized at steady state and during gene induction ^52,53^, the impact of transcriptional repression on bursting remains largely unexplored. To determine how IKZF1 controls transcription, we performed nascent RNA-FISH using intron probes for the fast-repressed genes *Ccnd2, Endod1, Myc*, and *Plekho2* to measure the number and intensity of transcriptional bursts in pre-B cells before (0h) and 1h after IKZF1-induction (Supplementary Fig. 3M). For all genes tested, the fraction of alleles bursting (Fig. 3H) was significantly decreased after 1h of IKZF1-induction, while the intensity of bursts remained largely unchanged (Fig. 3I). These data indicate that IKZF1 and NuRD induce repression primarily by reducing the probability of transcriptional bursting.

### Deregulation of fast-repressed genes in IKZF1 DN B-ALL

**Figure 4:**
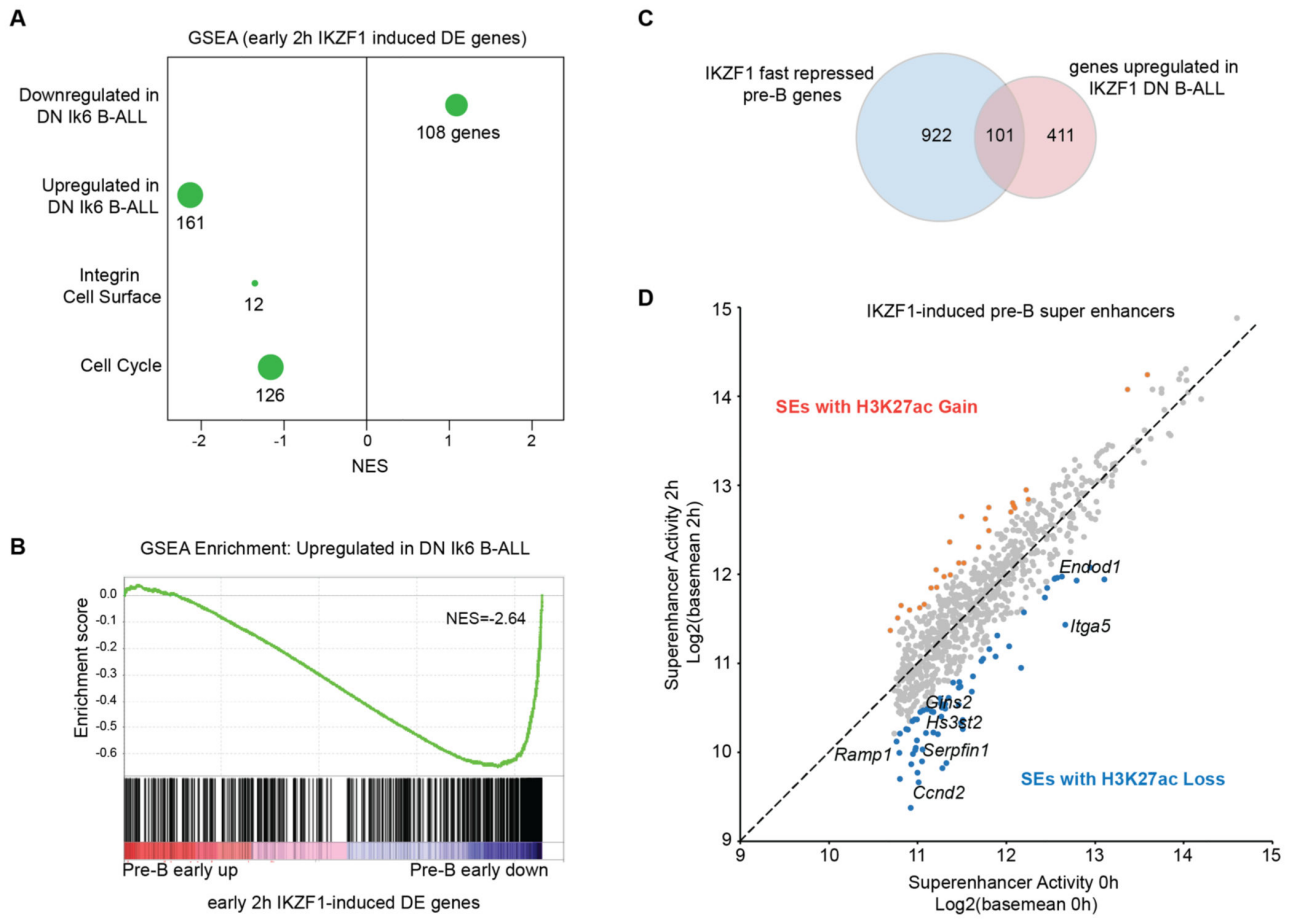
Deregulation of fast-repressed genes in IKZF1 DN B-ALL A. GSEA analysis of early (2h) IKZF1-induced transcriptional changes in pre-B cells with gene sets for deregulated genes in *IKZF1*-mutated DN Ik6 B-ALL, Integrin and Cell Surface Interactions (Reactome pathway) and Cell Cycle (Reactome pathway), with NES, and number of genes indicated. GSEA FDR q-values are 0.05 for Downregulated in DN Ik6 B-ALL, 0.00 for Upregulated in DN Ik6 B-ALL, 0.04 for Integrin and Cell Surface Interactions, and 0.2 for Cell Cycle. B. GSEA enrichment plot of early (2h) IKZF1-induced transcriptional changes in pre-B cells compared with the gene set of aberrantly upregulated genes in DN Ik6 B-ALL. C. Overlap of IKZF1 Fast Repressed pre-B genes and genes aberrantly upregulated genes in DN Ik6 B-ALL. D. Super-enhancer-associated IKZF1 Fast Repressed pre-B genes that are aberrantly upregulated in *IKZF1*-mutated DN IK6 B-ALL, and the degree of H3K27ac change 2h after IKZF1 induction, where super-enhancers with greater than 1.5-fold increase in H3K27ac and padj <0.01 in orange, and greater than 1.5-fold decrease and padj <0.01 in H3K27ac in blue.

To assess the relevance of IKZF1-regulated genes identified in B3 cells to B-ALL, we compared fast-repressed genes identified here with genes deregulated in IKZF1 DN-mutant B-ALL, where expression of DN IKZF1 results in increased expression of adhesion/integrin and cell cycle genes^33^. Although the B3 cell line was originally derived from a lymphoma rather than B-ALL model, the B3 gene expression profile closely resembles that of pre-B progenitors ^34,54^. Both integrin and cell cycle pathways pathways were enriched among our early-repressed genes, suggesting they are direct targets of IKZF1 repression (Fig. 4A). Strikingly, there was substantial overlap between the fast-repressed genes identified here and genes upregulated in IKZF1 DN-mutant B-ALL (Fig. 4AB, Supplementary Table 2). 20% of genes aberrantly upregulated in IKZF1 DN B-ALL were IKZF1 fast-repressed genes, and often associated with early-repressed pre-B SEs (*Itga5, Endod1, Ramp1, Serpinf1, Gins2, Hs3st2 and Hs3st3b1*, Fig. 4CD). Hence, genes identified here as direct targets of early repression by IKZF1 and NuRD in pre-B cells are critically dependent on IKZF1 for repression, and become aberrantly expressed in IKZF1-mutated leukemia.

### Conserved helical motifs in IKZF1 IDR are required for repression

**Figure 5:**
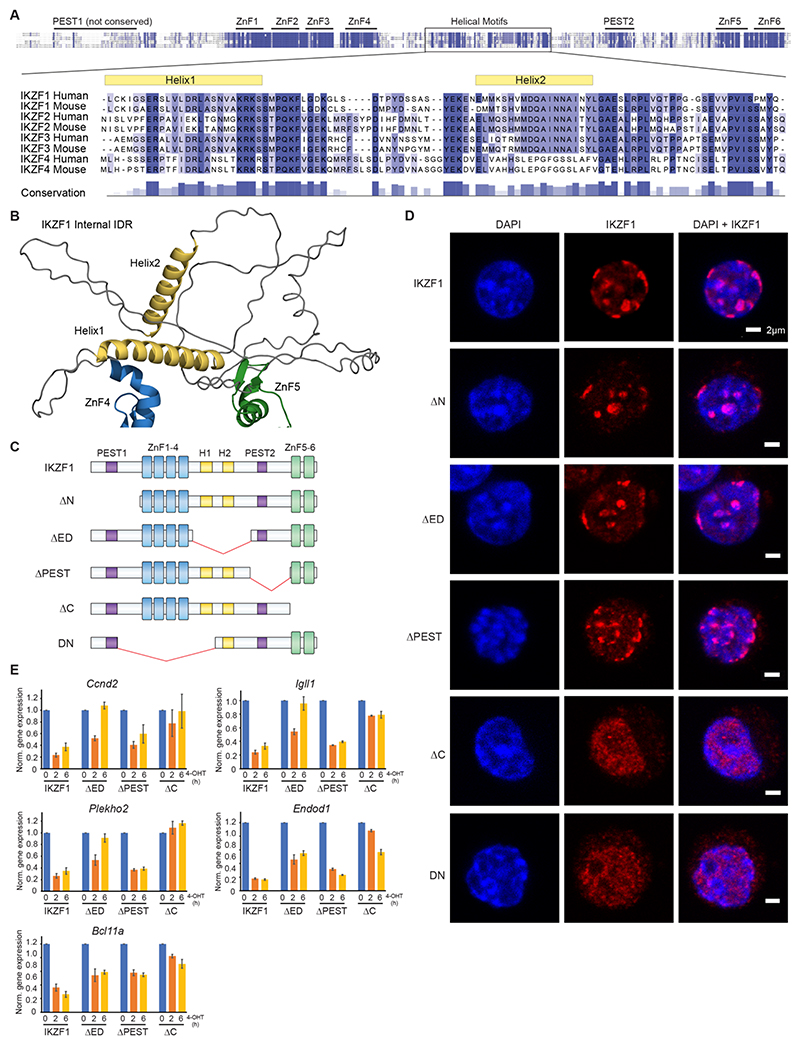
Conserved helical motifs in the IKZF1 IDR are required for gene repression A. Sequence alignment of mouse and human IKZF1-4 proteins, with magnified view of the α-helix-forming residues (yellow) in the internal IKZF intrinsically disordered region between ZnF4 and ZnF5. The level of conservation is shown in blue. B. AlphaFold structure of IKZF1, showing the internal disordered region between ZnF4 (blue) and ZnF5 (green), with the conserved helical motifs (Helix1 and Helix2) in yellow. C. Schematic of IKZF1 and truncation mutants, ΔN (N-terminus IDR deletion), ΔED (effector domain helical region IDR deletion), ΔPEST (proline, glutamic acid, serine, threonine-rich sequence IDR deletion), ΔC (C-terminus ZnF 5-6 deletion), DN (dominant negative Ik6). D. Immunofluorescence of B3 cells expressing HA-tagged IKZF1 WT and truncation mutants ΔN, ΔED, ΔPEST, ΔC, and DN. E. qPCR of normalized gene expression at several Fast Repressed genes before and 2h and 6h after induction with wildtype IKZF1 or mutants ΔED, ΔPEST and ΔC. Three biological replicates were performed. Primers can be found in Supplementary Methods.

To map residues within IKZF1 that confer repressive function we focused on intrinsically disordered regions (IDRs). Like most TFs, large parts of IKZF1, namely the N-terminal IDR and internal IDR, are largely unstructured (Supplementary Fig. 4A). PEST sequences are known to regulate IKZF1 stability^37^, but otherwise the functional significance of residues within the IDRs remains poorly defined. Using the TF prediction tool 9aaTAD (Ref^55,56^) we identified three α-helix-forming motifs in the IKZF1 internal IDR: SLVLDRLAS in Helix 1 and DMMTSHVMD and QAINNAINY in Helix 2 (Fig. 5AB, Supplementary Fig. 4B). Short helical motifs in eukaryotic TFs often govern important cofactor interactions^55,56^. These helical motifs as well as residues within adjacent loops are highly conserved in IKZF TFs (Fig. 5A).

The region overlapping Helix2 was previously identified as a transactivation-domain by yeast-one-hybrid assays^57^, but did not show activating function in mammalian cells^58^. We thus asked if the helical motifs mediated repression instead. We first established that IKZF1 truncation mutants in the conserved region containing the helical motifs (ΔED, effector domain) or other IDRs (ΔN, ΔPEST) retained DNA-binding ability, visualized as IKZF1 foci at DAPI-dense pericentromeric repeats known to be enriched for IKZF1-binding (Fig. 5CD, Supplementary Fig. 4C). Induction of wildtype IKZF1 repressed IKZF1-target genes at 2h, which was sustained at 6h (Fig. 5E). As a negative control, ΔC, defective in DNA-binding, lacked repressive function (Fig. 5E). Compared to wildtype IKZF1, ΔED mediated incomplete repression at 2h and especially 6h (Fig. 5E). Deletion of Helix1 (ΔED1) or Helix2 (ΔED2) in isolation led to reduced repression (Supplementary Fig. 4CDE). By contrast, ΔPEST which contains the helical motifs but lacks the adjacent PEST region showed near wild-type repression, supporting a specific role for the conserved helical motifs in sustained repression of IKZF1 target genes (Fig. 5E).

### IKZF1 helical motif region mediates NuRD interaction

**Figure 6:**
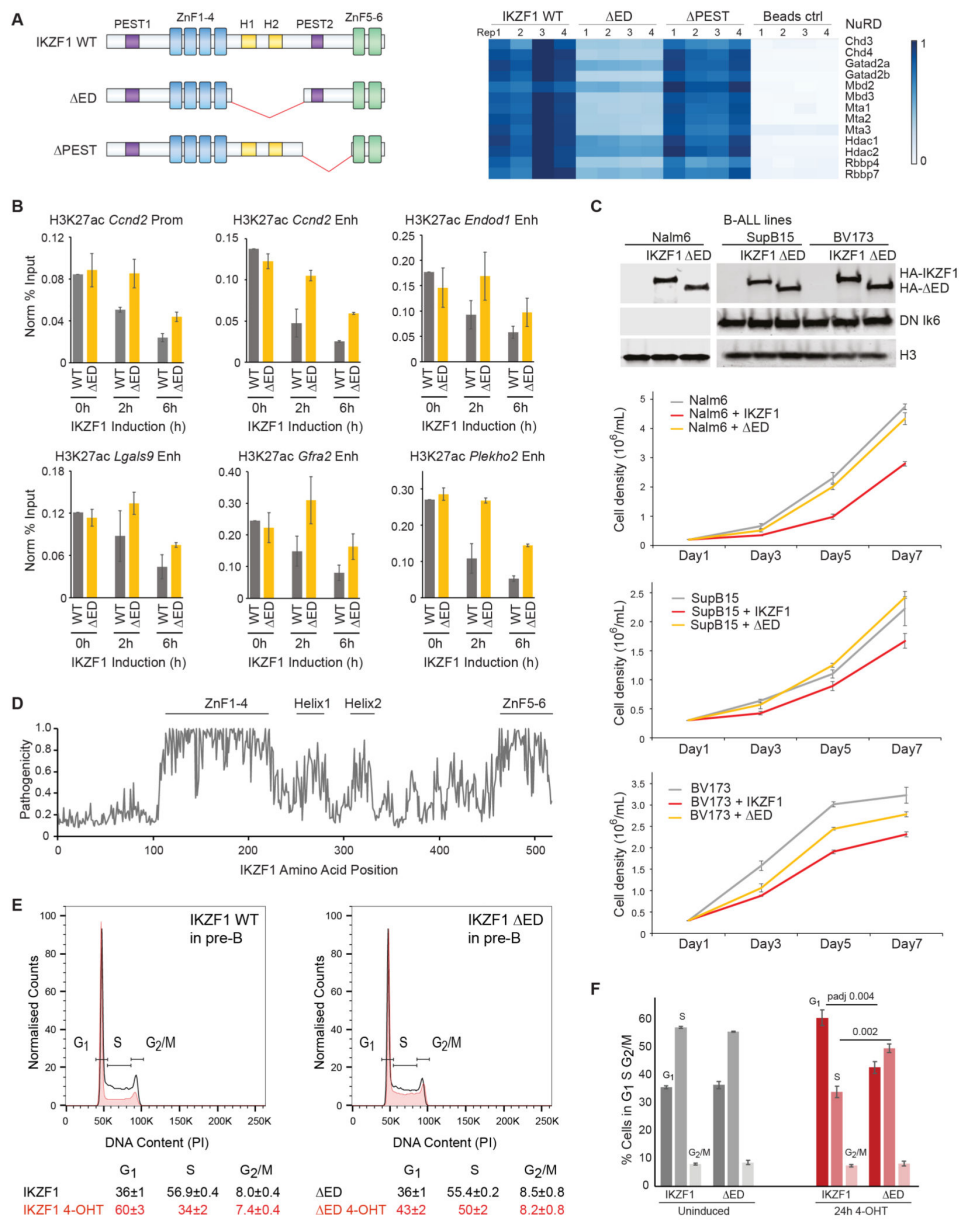
Conserved helical motifs are required for NuRD-interaction and anti-proliferative function of IKZF1 A. Heatmap of relative NuRD-interaction in AP-MS of IKZF1 WT, ΔED, ΔPEST, and control cells. Four replicates were performed per condition. B. H3K27ac levels at IKZF1 Fast Repressed gene promoters and enhancers before (0h) and 2h and 6h after induction with IKZF1 WT or ΔED. ChIP-qPCR was performed in triplicate for all sites, and negative control sites are shown in Supplementary Fig. 5B. Primers can be found in Supplementary Methods. C. Western blot of human B-ALL lines Nalm6, SupB15, and BV173 transduced with equivalent levels of inducible IKZF1 or ΔED (top panel), and growth curve over seven days following induction with 4-OHT (bottom panels). SupB15 and BV173 express the IKZF1 DN Ik6 isoform. Three replicates were performed. D. AlphaMissense mean pathogenicity score by amino acid position in the IKZF1 protein. Positions of ZnF1-6 and helical motifs are indicated. E. Cell cycle analysis of pre-B cells transduced with IKZF1 WT or ΔED without induction (black) and 24h after induction with 4-OHT performed in triplicate with one representative replicate shown. The proportion of cells in G1, S, and G2/M were determined by fitting with the Watson Pragmatic Model. Three replicates were performed. F. The proportion of cells in G1, S, and G2/M in pre-B cells transduced with IKZF1 WT or ΔED without induction (black) and 24h after induction with 4-OHT performed in triplicate. Pairwise t-tests were performed between IKZF1 WT and ΔED, with the Bonferroni corrected p-adjusted value stated.

To explore the mechanism through which the helical motif region contributes to IKZF1 repressive and anti-proliferative functions, we performed affinity-purification MS of wildtype IKZF1, silencing-defective ΔED, and silencing-competent ΔPEST. While most interactors were preserved in the truncation mutants (Supplementary Fig. 5A), ΔED (but not ΔPEST) showed substantially reduced interactions with NuRD subunits (Fig. 6A, Supplementary Fig. 5B), demonstrating that the conserved helical motifs mediate NuRD-binding. In accord with reduced NuRD-interaction, the silencing-defective ΔED also showed weaker ability to reduce H3K27ac levels at target genes compared to wildtype IKZF1 (Fig. 6B, Supplementary Fig. 5C).

### Helical motifs mediate the anti-proliferative function of IKZF1 in human B-ALL cell-lines

To further assess the role of this NuRD-interacting region, we tested the requirement for the conserved helical motifs for the growth-suppressive ability of IKZF1 in human B-ALL cells. IKZF1 is a tumour suppressor in acute pre-B and T-cell leukemia, and exhibits anti-proliferative effects through repression of *Myc* and cell cycle genes^59^. Several cycle cell genes (*Ccnb1, Ccnd1, Ccnd2*) were IKZF1 fast-repressed targets in pre-B cells, among which cyclins *Ccnd1/2* are critical for the G1 to S transition. As expected^60,61^, wildtype IKZF1 reduced the growth of B-ALL line Nalm-6, and SupB15 and BV173 which express DN IKZF1(Fig. 6C). Compared to wildtype IKZF1, ΔED showed reduced anti-proliferative effects, indicating that the conserved helical region is required for the anti-proliferative functions of IKZF1 in human B-ALL cells (Fig. 6C). Strikingly, mutations in the helical motif region are predicted to be highly pathogenic by AlphaMissense, with pathogenicity scores only secondary to mutations in ZnF regions (Fig 6D).

### Helical motifs are required for IKZF1-mediated G1-arrest

To analyze the effect of IKZF1’s anti-proliferative functions more quantitatively, we performed cell cycle analysis in pre-B cells transduced with wildtype IKZF1 and ΔED (Supplementary Fig. 5D). Induction of wildtype IKZF1 for 24h led to an increase in the proportion of cells arrested in G1 (60%) (Fig. 6EF). Notably, this anti-proliferative function of IKZF1 was attenuated upon deletion of the conserved helical region ΔED (43% in G1) (Fig. 6EF). As pre-B cells express the paralog IKZF3, which could conceivably complicate the interpretation of this experiment, we repeated this experiment in 3T3 fibroblasts (Supplementary Fig. 5EFG). Induction of wildtype IKZF1 resulted in an almost complete arrest of 3T3 cells in G1 by 24h. By contrast, the anti-proliferative effect of ΔED was markedly reduced. These data show that the helical motifs which mediate NuRD-interaction are required for full transcriptional repression and anti-proliferative functions of IKZF1.

### Structural basis for IKZF1-NuRD interaction

**Figure 7:**
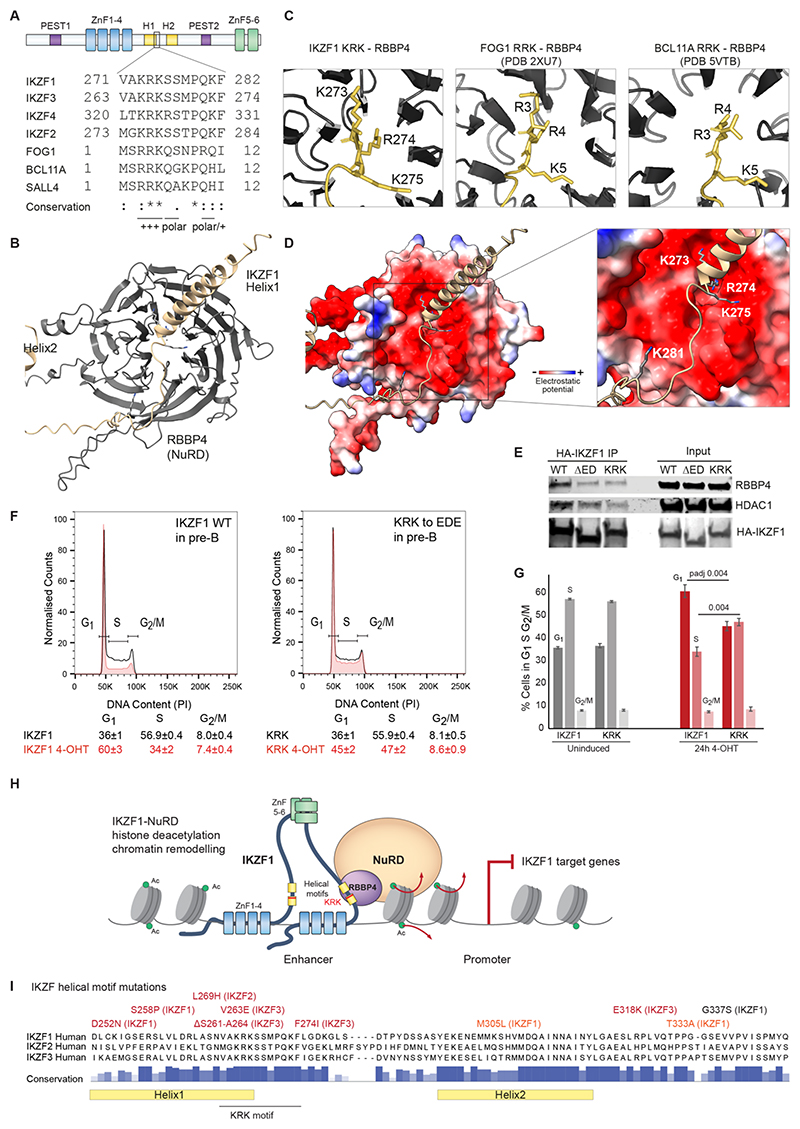
Structural basis of IKZF1 and NuRD interaction A. Protein sequence alignment of the “KRKSSMPQKF” motif present in Helix1 of IKZF family with the matching N-terminal motif found in NuRD-interacting TFs FOG1, BCL11A, SALL4. Conservation and residue properties are shown below the alignment. B. AlphaPulldown model of the interaction interface between IKZF1 Helix1 with the NuRD subunit RBBP4. Interface pTM score of 0.73. C. Close up of the KRK residues in IKZF1, FOG1 (PDB 2XU7), and BCL11A (PDB 5VTB) within the central pocket of RBBP4. D. Model of the IKZF1 Helix1 - RBBP4 interaction showing the positive KRK (residues 273-275) at the end of Helix1 positioned within the negatively charged (red) central pocket of RBBP4. The flexible loop (residues 276-281) binds along the negative charged groove between two β-propeller blades of RBBP4. E. Immunoprecipitation of HA-IKZF1 WT, ΔED, and KRK by HA-beads from nuclear extract, and immunoblot detection of NuRD subunits RBBP4 and HDAC1. F. Cell cycle analysis of pre-B cells transduced with IKZF1 WT or KRK to EDE charge-reversal mutant without induction (black) and 24h after induction with 4-OHT performed in triplicate with one representative replicate shown. The proportions of cells in G1, S, and G2/M were determined by fitting with the Watson Pragmatic Model. G. The proportion of cells in G1, S, and G2/M in pre-B cells transduced with IKZF1 WT or KRK without induction (black) and 24h after induction with 4-OHT performed in triplicate. Pairwise t tests were performed between IKZF1 WT and KRK, with the Bonferroni corrected p-adjusted value stated. H. IKZF1-NuRD mediates histone deacetyation and chromatin remodelling at target gene promoters and enhancers to mediate transcriptional repression. Mechanistically, interaction between IKZF1 and NuRD is mediated through the helical motifs in the internal IDR of IKZF1 binding to the RBBP4 subunit. I. Missense mutations in the conserved helical motif region of *IKZF1, IKZF2*, and *IKZF3* detected in patients with hematopoietic malignancies, including pediatric B-ALL, T-ALL, Hairy cell leukemia, Hodgkin lymphoma, Diffuse large B-cell lymphoma, and Chronic myeloid leukemia. Five computational methods, Polyphen2, AlphaMissense, Fathmm, MutPred2, and Panther were used to predict the functional impact of each missense mutations - mutations in red were predicted to be pathogenic by at least 3 models, mutations in orange by 1-2 models, and mutations in black were predicted to be benign by all. Prediction scores and references can be found in Supplementary Fig. 6H and Supplementary Table 3.

Closer inspection revealed the residues KRKSSMPQKF in Helix1 and the adjacent loop match a sequence found in the zinc finger TFs BCL11A, FOG1, and SALL1-4 (Fig. 7A). This motif mediates interactions between these TFs and the NuRD subunit RBBP4 (Ref.^62–65^), the most abundant NuRD subunit identified by IKZF1 ChIP-MS (Supplementary Fig. 6A). This KRK motif is conserved in IKZF family TFs across all deuterostome phyla^66^, but had not previously been functionally characterized. A high-confidence model of the IKZF1 helical motifs and RBBP4 generated by AlphaPulldown reveals an interaction interface that is structurally identical to the interaction of FOG1 and BCL11A with RBBP4 (Ref.^62–64^ Fig. 7BC, Supplementary Fig. 6B). The positively charged KRK at the tip of Helix1 inserts into the negative central pocket of RBBP4, and the flexible loop after Helix1 interacts with the groove between two β-propeller blades in RBBP4 (Fig. 7D). A charge-reversal mutation of the KRK residues to EDE reduced the interaction with NuRD, similar to deletion of the helical motif region (Fig. 7E). Furthermore, the KRK mutation attenuated the anti-proliferative function of IKZF1 in pre-B and 3T3 cells, again to a similar extent as the deletion of the helical motif region (Fig. 7FG, Supplementary Fig. 6CDEF). Hence, the highly conserved KRK motif in IKZF1 mediates IKZF1-NuRD-interactions through a mechanism shared with other Zn finger TFs.

## Discussion

Pioneering studies have established IKZF1 as a key regulator of lymphocyte development^2–4^, and an important tumor suppressor in B-ALL (Ref.^5–8^). Based on time-resolved analyses, we find that the immediate response to IKZF1 binding in pre-B cells comprises loss of chromatin accessibility and H3K27ac at regulatory elements and transcriptional repression. Fast-repressed genes were enriched in pathways related to lymphocyte differentiation, and associated with a greater number of enhancers, which exhibited faster chromatin state changes in response to IKZF1 than promoters. Analysis of transcriptional burst parameters revealed that IKZF1 represses target genes primarily through reducing the probability of transcriptional bursting, rather than burst intensity.

We did not observe direct chromatin-opening or activating function of IKZF1 in pre-B cells. Sites with early-increased accessibility were depleted of IKZF1 binding, and transcriptional activation was temporally delayed relative to repression. Accordingly, corepressors by far outweighed coactivators by proteomic quantification. Previously reported interactions with coactivators including p-TEFb (Ref.^25,26^) and SWI/SNF (Ref.^22–24^) were quantitatively eclipsed by corepressors. KAP1, which interacts with KRAB ZnFs to facilitate H3K9me3 (Ref.^49,67^) was a highly-abundant novel interactor. Although dispensable for the initiation of IKZF1-mediated repression, further exploration may reveal cooperation between IKZF1 and heterochromatin-associated factors in maintaining the silenced state.

Dissection of IDRs identified highly conserved helical motifs that mediate IKZF1-NuRD interaction, contribute to target gene silencing, and attenuate the proliferation of IKZF1-mutant human B-ALL cells (Fig. 7H). We find a conserved sequence (R/K)RKXXXPQ present in IKZF1 Helix1 that is shared with other NuRD-binding ZnF TFs FOG1, BCL11A, and SALL4. Interestingly, while this motif is in the internal IDR of IKZF1, it is N-terminal in the other ZnFs. Helix1 therefore likely provides the structural rigidity necessary for insertion of the KRK residues into RBBP4. How Helix2 supports the interaction between IKZF1 and NuRD remains to be determined. Sumoylation sites are a feature of TF repression domains^68^, and sumoylation of IKZF1 can disrupt interactions with NuRD (Ref.^38^). IKZF1 K240 adjacent to Helix1 is one of three IKZF1 sumoylation sites^38,69^, raising the strong possibility that post-translational mechanisms may target conserved helical motifs to modulate IKZF1-NuRD interactions.

The impact of missense variants within the helical domains are predicted to be highly pathogenic (Fig 7I, Supplementary Fig. 6G, Supplementary Table 3). Missense mutations in the IKZF1 helical motif region have been identified in T-ALL (Ref.^70^) and pediatric B-ALL (Ref.^71^). Mutations in the corresponding region in IKZF2 and IKZF3 have been similarly found in other hematopoietic neoplasms^72–75^. This suggests that disruption of this region which confers NuRD binding and repressive function may contribute to leukemogenesis. Finally, we find substantial overlap between early repressed genes identified here and aberrantly upregulated genes in IKZF1-mutated B-ALL, and several IKZF1-repressed super enhancer associated genes *ITGA5, CCND2, GFRA2, BCL11A* have been implicated in multiple cancers^76–80^. This indicates that a large number of genes aberrantly expressed in *IKZF1*-mutated leukemia are highly sensitive to IKZF1 dosage, and dependent on IKZF1/NuRD for repression. The molecular insights uncovered for IKZF1-mediated transcriptional repression in pre-B cells are therefore likely to inform mechanisms of deregulation in leukemia.

## Supplementary Material

Supplemental Figures Methods

Supplemental Table1

Supplemental Table2

Supplemental Table3

## Data Availability

Proteomics data generated for this study have been deposited in PRIDE PXD050989 and NGS data in GEO under accession number GSE256299.

## Key Points

- IKZF1 and NuRD induce rapid loss of chromatin accessibility and H3K27ac at enhancers and super-enhancers to repress target genes
- Immediate IKZF1 target genes in pre-B cells significantly overlap with deregulated genes in *IKZF1-*mutated B-ALL
