# Supplementary material for: Conserved helical motifs in the IKZF1 disordered region mediate NuRD interaction and transcriptional repression
Source: Blood. Author manuscript; Available in PMC 2025 Mar 11. (PMC7617475; doi:10.1182/blood.2024024787)
Supplement: Supplemental Figures Methods [file EMS203634-supplement-Supplemental_Figures_Methods.pdf]

Conserved helical motifs in the IKZF1 disordered region mediate NuRD interaction and transcriptional repression

Supplementary Table 1 – IKZF1 RNAseq DESeq2.xlsx
Supplementary Table 2 – Up in DNIk6 BALL.xlsx
Supplementary Table 3 – IKZF123 cancer mutations.xlsx

Supplementary Figures and Methods

Supplementary Figure 1

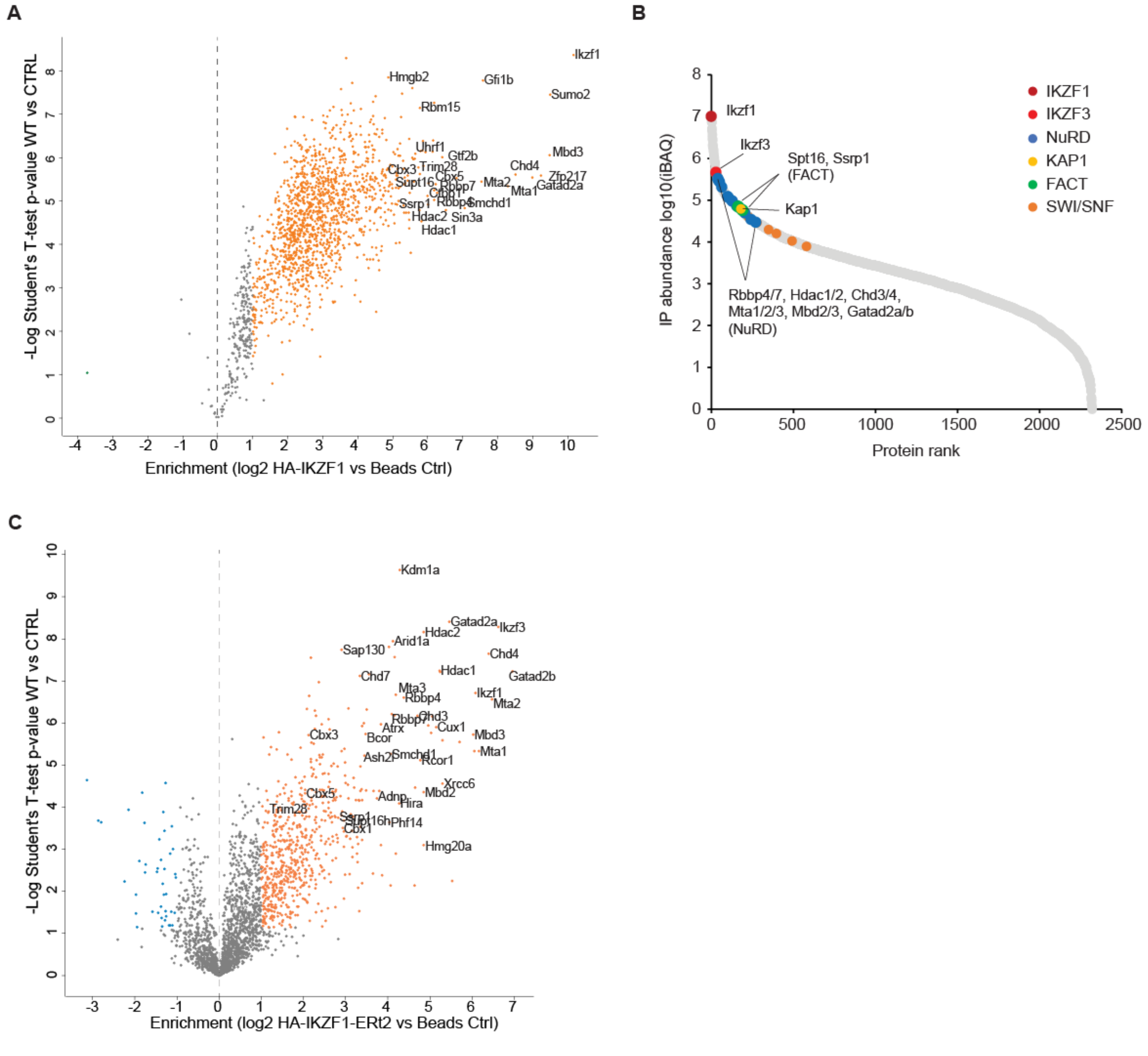

Supplementary Figure 1. IKZF1 chromatin-mediated and in-solution interactors

A. Volcano plot of proteins identified in anti-HA ChIP-MS of HA-IKZF1 expressing B3 cells vs. untransduced B3 control. Average of three replicates.
B. IKZF1-ERt2 (2h 4-OHT induction) affinity purification MS interactors performed in absence of crosslinking displayed by abundance, with IKZF1 in maroon, IKZF3 (Aiolos) in red, NuRD subunits in blue, KAP1 in yellow, FACT subunits in green, and SWI/SNF subunits in orange. Average of four replicates.
C. Volcano plot of proteins identified in anti-HA affinity purification MS of HA-IKZF1-ERt2 expressing B3 cells (2h 4-OHT induction) compared to untransduced B3 control.

Supplementary Figure 2

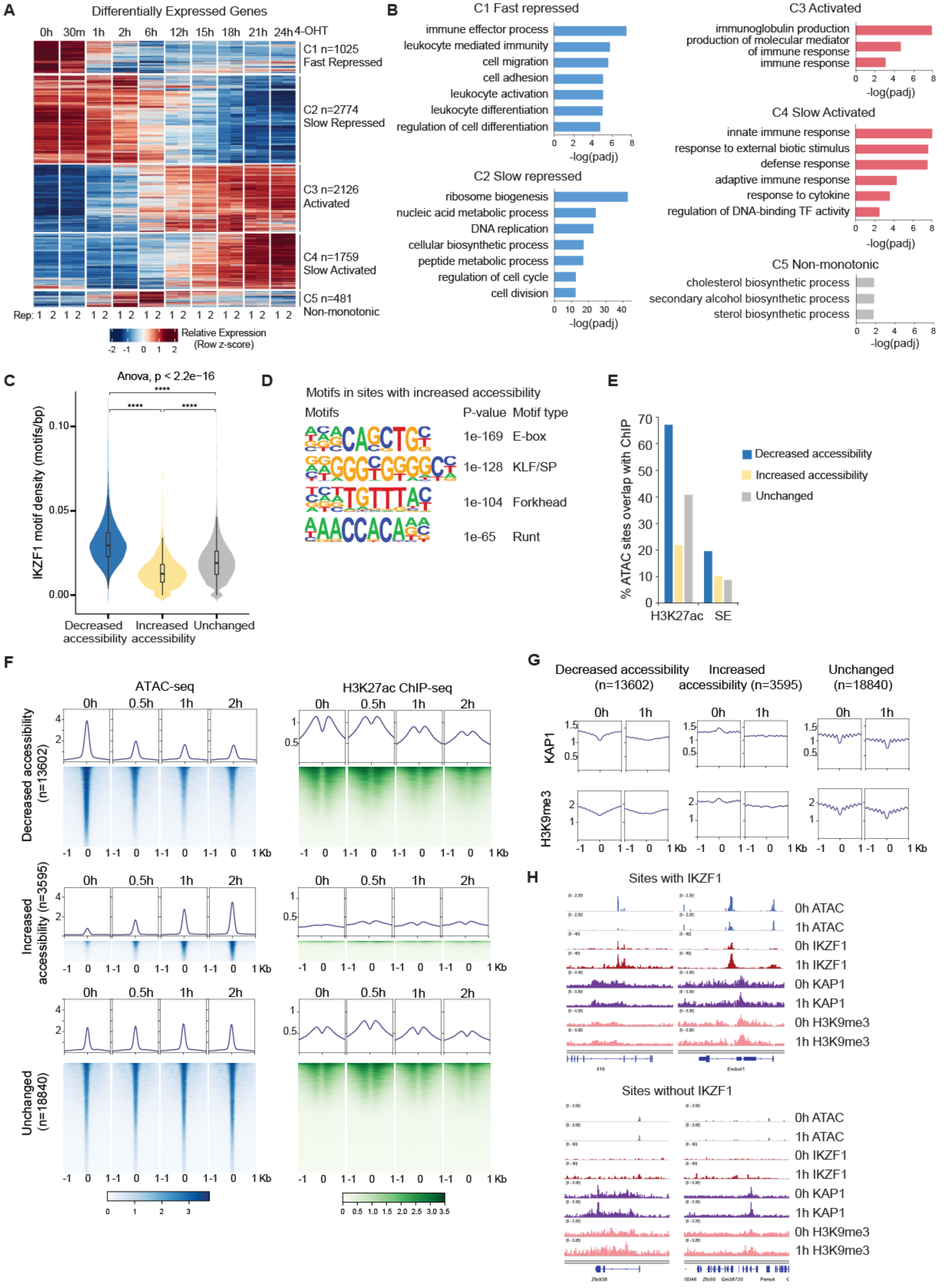

Supplementary Figure 2. IKZF1 induces rapid loss in chromatin accessibility, H3K27ac, and gene repression

A. Heatmap of differentially expressed genes (padj <0.01 and log2FC>1 or <-1) over a nascent chromatin-associated RNA-seq 24h timecourse following IKZF1 induction performed in duplicate, and K-means clusters of genes by behaviour into C1 Fast Repressed, C2 Slow Repressed, C3 Activated, C4 Slow Activated, and C5 non-monotonic.
B. Gene ontology of top pathways enriched in C1 Fast Repressed, C2 Slow Repressed, C3 Activated, C4 Slow Activated and C5 non-monotonic genes.
C. IKZF1 motif density (GGAA and GGGA count/bp) at sites with early decreased accessibility, increased accessibility, and unchanged accessibility.
D. Homer motif analysis showing top enriched motifs at ATAC NFR sites with early increased accessibility compared to regions with unchanged accessibility.
E. Overlap of sites with early decreased accessibility, increased accessibility or with unchanged accessibility with H3K27ac peaks and ROSE called superenhancers based on H3K27ac ChIP-seq.
F. Heatmap and metaprofile plot showing chromatin accessibility and H3K27ac at 0hm 30m, 1h, and 2h following IKZF1 induction +/-1kb of ATAC peaks with early decreased, increased or unchanged accessibility.
G. Metaprofile plot showing KAP1 binding and H3K9me3 levels at 0h and 1h following IKZF1 induction +/-1kb of ATAC peaks with early decreased, increased or unchanged accessibility.
H. IGV browser tracks showing KAP1 (purple) and H3K9me3 (pink) at genomic regions with IKZF1-ERt2 binding (red) and decreased accessibility ATAC (blue) and at genomic regions without IKZF1 binding.

Supplementary Figure 3

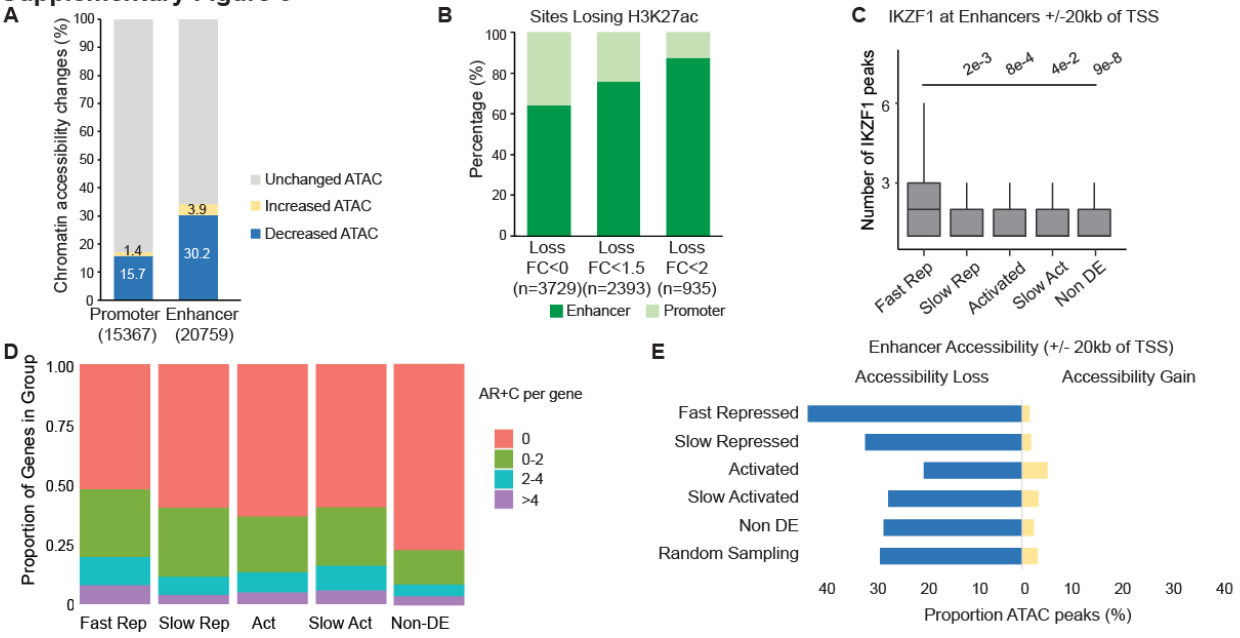

F

	Accessibility loss		Accessibility gain	
	Odds Ratio	Padj.	Odds Ratio	Padj.
Fast Repressed	1.90	<0.0001	0.47	0.01
Slow Repressed	1.16	n.s.	0.58	n.s.
Activated	0.61	<0.0001	1.68	0.03
Slow Activated	0.92	n.s.	1.06	n.s.

G

	H3K27ac loss		H3K27ac gain	
	Odds Ratio	Padj.	Odds Ratio	Padj.
Fast Repressed	2.94	<0.0001	0.25	0.0002
Slow Repressed	1.12	n.s.	0.66	n.s.
Activated	0.37	<0.0001	3.60	<0.0001
Slow Activated	1.07	n.s.	1.57	0.02

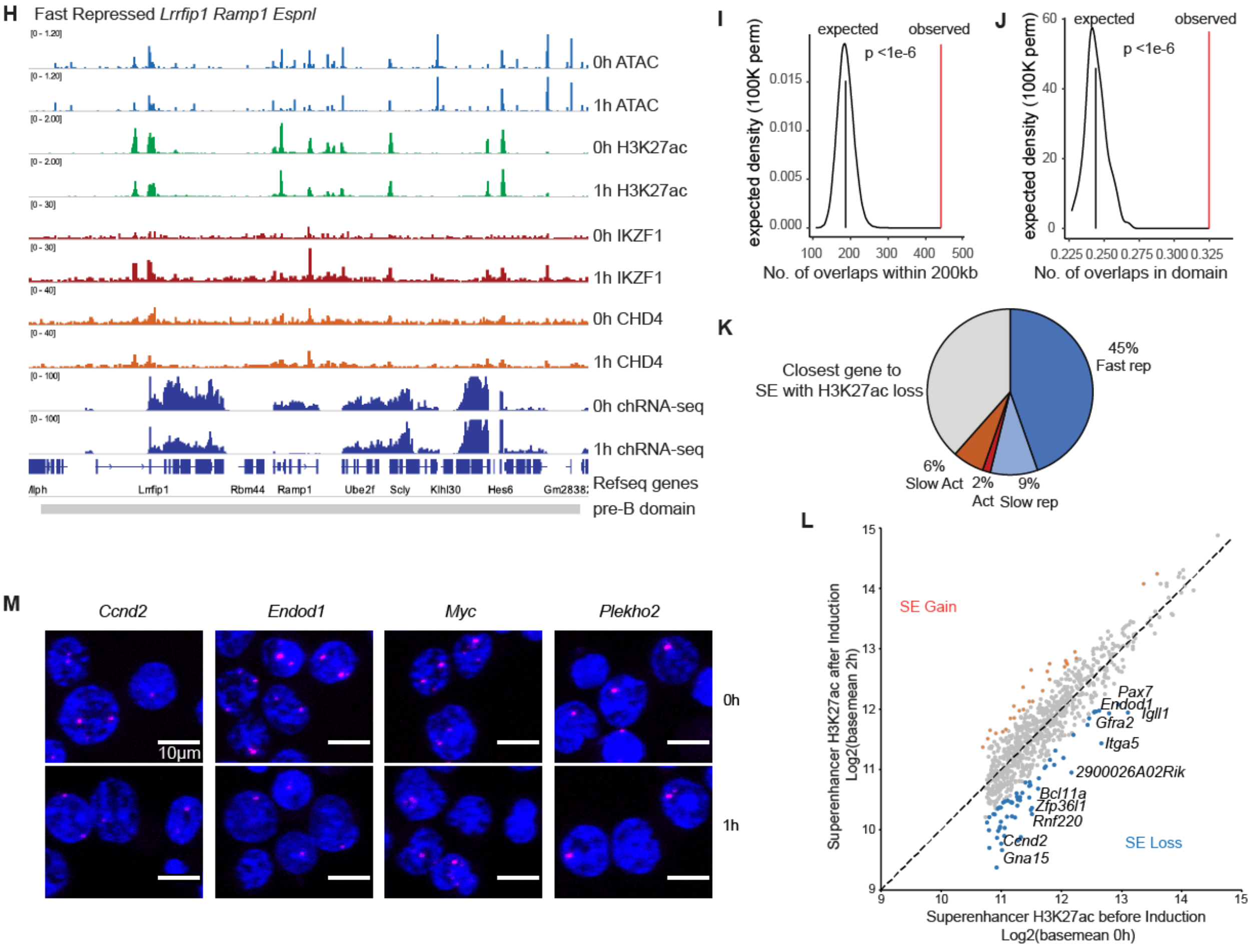

Supplementary Figure 3. IKZF1-repressed targets are associated with repressed enhancers and super enhancers

A. Proportion of sites that show early decreased or increased chromatin accessibility at active promoters vs enhancers.
B. Proportion of enhancers (dark green) and promoters (light green) at sites showing significant loss of H3K27ac (padj<0.01) and greater than 1.5 fold and 2 fold loss of H3K27ac.
C. Number of IKZF1 peaks at enhancers +/- 20kb TSS of Fast Repressed, Slow Repressed, Activated, Slow Activated, and non-differentially expressed genes. Pairwise Wilcoxon rank sum test was performed for Fast Repressed vs other gene class with Bonferroni corrected p-adjusted values stated.
D. Proportion of Enhancer – Promoter connections for Fast Repressed, Slow Repressed, Activated, Slow Activated, and non-differentially expressed genes, where AR is the correlation between enhancer and promoter activity and C is a measure of contact frequency between the enhancer and promoter (see supplemental Methods)
E. Proportion of enhancer ATAC peaks within +/- 20kb of TSS Fast Repressed, Slow Repressed, Activated, Slow Activated, non-differentially expressed, and randomly sampled genes that show significant (padj<0.01) decreased or increased accessibility within 2h of IKZF1 induction.
F. Odds ratio and significance for enhancer accessibility loss or gain of Fast Repressed, Slow Repressed, Activated or Slow Activated genes compared to a randomly sampled set of genes.
G. Odds ratio and significance for enhancer H3K27ac loss or gain of Fast Repressed, Slow Repressed, Activated or Slow Activated genes compared to a randomly sampled set of genes.
H. IGV browser of locus with three Fast Repressed genes *Lrrfip1, Ramp1, Espnl* with tracks at 0h and 1h after IKZF1 induction for ATAC in blue, H3K27ac in green, IKZF1-ERt2 in red, CHD4 in orange, chRNA-seq and Refseq genes in navy, and pre-B cell domains in grey.
I. Number of observed overlaps (red line) between Fast Repressed genes within <200kb of each other versus number of overlaps (black) over 100000 runs with randomised gene identities.
J. Number of observed overlaps (red line) between Fast Repressed genes within the same pre-B topologically associated domain versus number of overlaps (black) over 100000 runs with randomised gene identities.
K. Proportion of Fast Repressed, Slow Repressed, Activated, Slow Activated genes neighbouring superenhancers with 1.5-fold decrease in H3K27ac (and padj<0.01) at 2h of IKZF1 induction.
L. Levels of H3K27ac at all SE before and 2h after IKZF1 induction, superenhancers with greater than 1.5-fold increase in H3K27ac and padj <0.01 in orange, and greater than 1.5-fold decrease and padj <0.01 in H3K27ac in blue. Fast Repressed genes are labelled next to their closest SE.
M. Representative images of intron RNA-FISH against IKZF1 repressed genes *Ccnd2, Endod1, Myc, Plekho2* before 0h and at 1h of IKZF1 induction.

Supplementary Figure 4

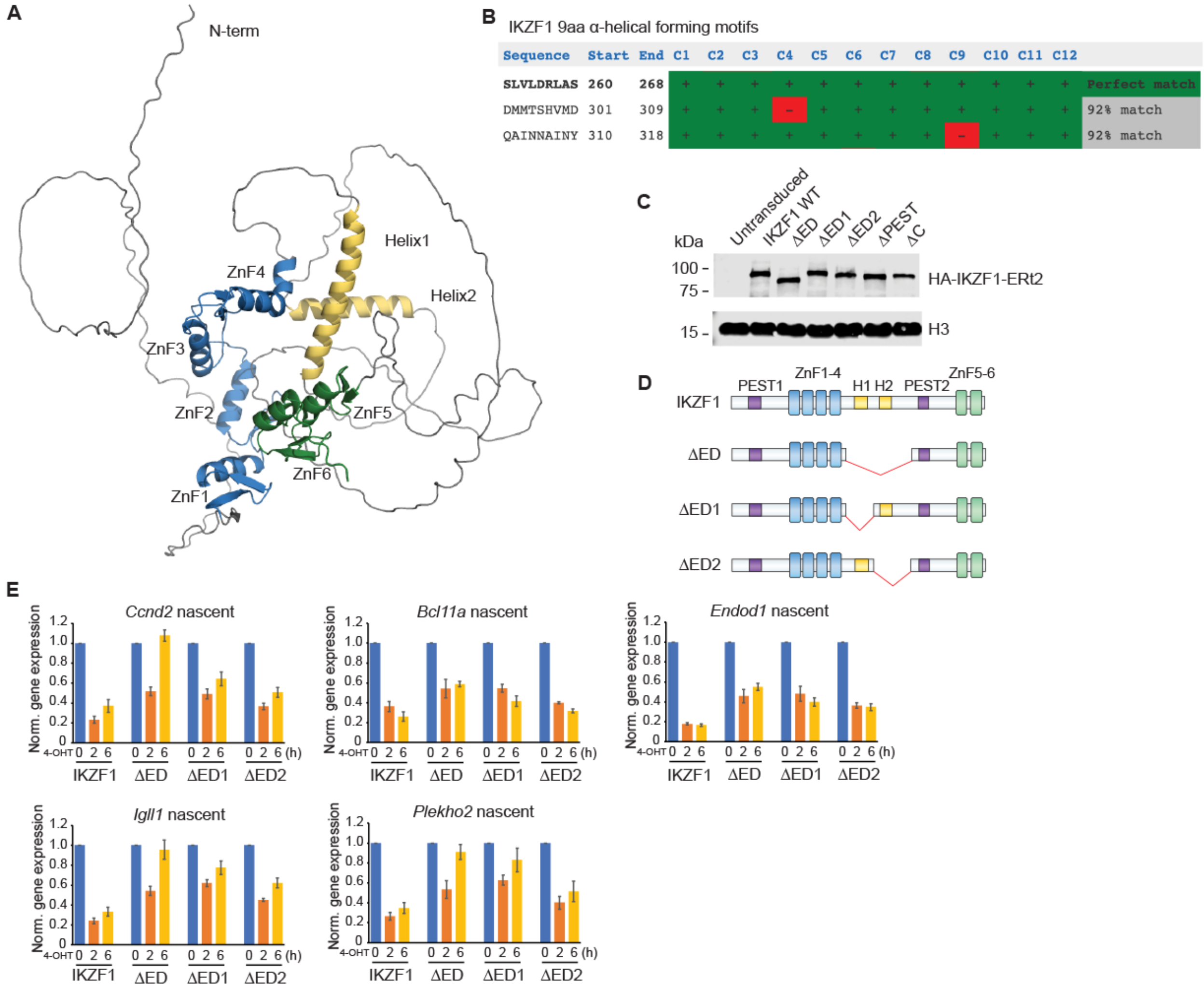

Supplementary Figure 4. Identification of helical motifs in internal IDR

A. AlphaFold structure of the IKZF1 protein with the conserved helical motifs in yellow, DNA binding ZnF1-4 in blue, and dimerization ZnF5-6 in green.
B. Top three 9aa motifs in the mouse IKZF1 protein obtained from the 9aaTAD Prediction Tool.
C. Western blot showing equivalent levels of IKZF1 and mutant expression in B3 cells.
D. Schematic of IKZF1 mutants ΔED, ΔED1 (Helix1 deletion), and ΔED2 (Helix2 deletion).
E. qPCR of normalised gene expression at several Fast Repressed genes before and after induction with wildtype IKZF1 or mutants ΔED, ΔED1, and ΔED2.

Supplementary Figure 5

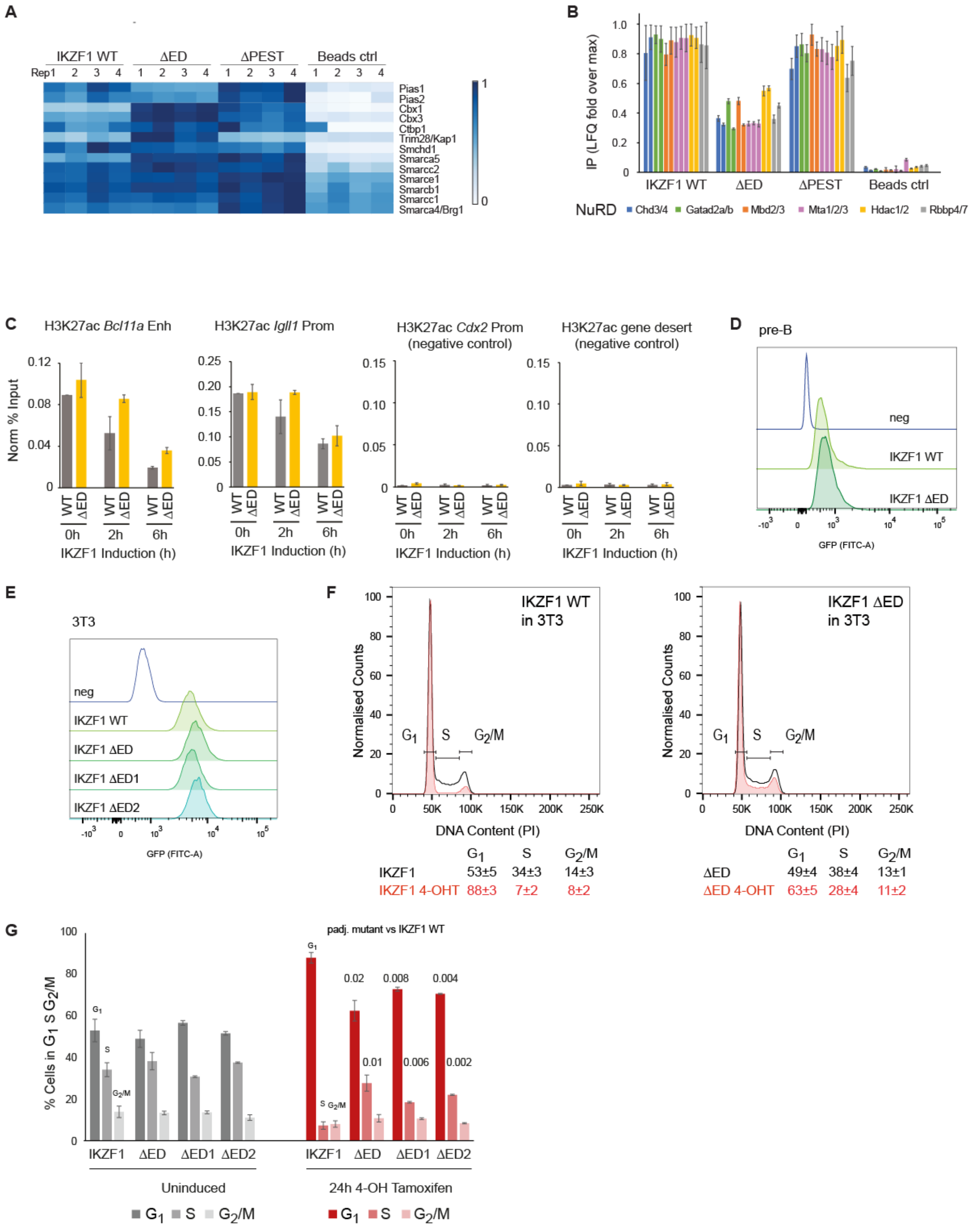

Supplementary Figure 5. Helical motif region mediates NuRD-interaction and is necessary for IKZF1-mediated repression

A. Heatmap of IKZF1 interactions from affinity purification-MS of IKZF1 WT, ΔED, ΔPEST, showing similar levels of interaction with PIAS E3-ligases, heterochromatin-associated proteins and SWI/SNF proteins. Four replicates were performed for each condition.
B. NuRD subunit abundance from affinity purification-MS of IKZF1 WT, ΔED, ΔPEST, and untransduced cells. Average of four replicates.
C. H3K27ac levels at IKZF1 Fast Repressed gene promoters and enhancers and two negative control sites before (0h) and 2h and 6h after induction with IKZF1 WT or ΔED. Three replicates were performed. Primers can be found in Supplementary Methods.
D. Flow cytometry histogram showing equivalent levels of IKZF1 WT and ΔED in transduced B3 cells.
E. Flow cytometry histogram showing equivalent levels of IKZF1 WT and mutants in transduced 3T3 cells.
F. Cell cycle analysis of 3T3 cells transduced with IKZF1 WT or ΔED without induction (black) and 24h after induction with 4-OHT performed in triplicate with one representative replicate shown. The proportion of cells in G1, S, and G2/M were determined by fitting with the Watson Pragmatic Model.
G. The proportion of cells in G1, S, and G2/M in 3T3 cells transduced with IKZF1 WT, ΔED, ΔED1, and ΔED2 without induction (black) and 24h after induction with 4-OHT performed in triplicate. Pairwise t-tests were performed between IKZF1 WT and mutants, with the Bonferroni corrected p-adjusted value stated.

Supplementary Figure 6

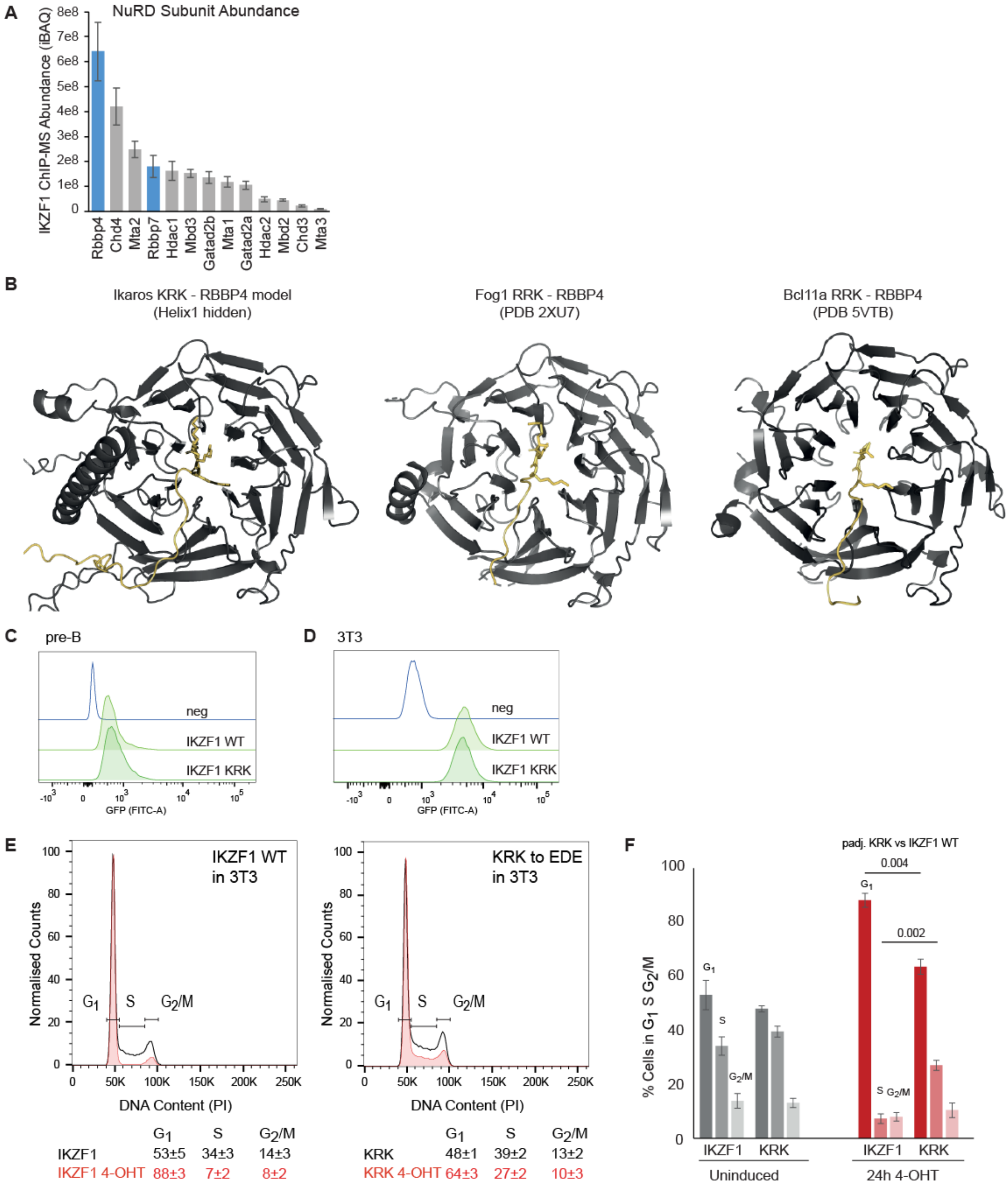

G

Gene	Mutation	Position	sample	Polyphen2	AlphaMissense	Fathmm	MutPred2	Panther
IKZF1	D252N	Start of Helix1	Pediatric B-ALL	pathogenic	ambiguous	tolerated	tolerated	pathogenic
IKZF1	S258P	Start of Helix1	Pediatric B-ALL	tolerated	tolerated	pathogenic	pathogenic	pathogenic
IKZF1	M305L	Start of Helix2	T-ALL	tolerated	tolerated	tolerated	tolerated	pathogenic
IKZF1	T333A	Loop after Helix2	Pediatric B-ALL	pathogenic	tolerated	tolerated	tolerated	pathogenic
IKZF1	G337S	Loop after Helix2	Pediatric B-ALL	tolerated	tolerated	tolerated	tolerated	tolerated
IKZF2	L269H	Middle of Helix1	Hairy cell leukemia	pathogenic	pathogenic	pathogenic	tolerated	pathogenic
IKZF3	V263E	Middle of Helix1	Hodgkin lymphoma	pathogenic	pathogenic	pathogenic	pathogenic	tolerated
IKZF3	del S261-264 **	Middle of Helix1	Diffuse large B cell lymphoma	pathogenic	pathogenic	pathogenic	pathogenic	pathogenic
IKZF3	F274I	Loop after Helix1	Diffuse large B cell lymphoma	pathogenic	pathogenic	tolerated	tolerated	tolerated
IKZF3	E318K	Loop after Helix2	Chronic myeloid leukemia	pathogenic	pathogenic	tolerated	pathogenic	tolerated

pathogenic / ambiguous / tolerated

** deletion most likely pathogenic due to high pathogenicity scores of individual residues

Supplementary Figure 6. IKZF1 binds RBBP4 through conserved KRK sequence shared with other NuRD-interacting TFs

A. Relative abundance (iBAQ) of IKZF1-associated NuRD subunits detected by ChIP-MS with RBBP4/7 in blue.
B. The interaction interface between IKZF1 Helix1 “KRKSSMPQKF” motif with RBBP4 generated by AlphaPulldown, and the crystal structures of the equivalent motif in FOG1 (PDB 2XU7) and BCL11A (PDB 5VTB) with RBBP4.
C. Flow cytometry histogram showing equivalent levels of IKZF1 WT and KRK in transduced B3 cells.
D. Flow cytometry histogram showing equivalent levels of IKZF1 WT and KRK in transduced 3T3 cells.
E. Cell cycle analysis of 3T3 cells transduced with IKZF1 WT or KRK without induction (black) and 24h after induction with 4-OHT performed in triplicate with one representative replicate shown. The proportion of cells in G1, S, and G2/M were determined by fitting with the Watson Pragmatic Model.
F. The proportion of cells in G1, S, and G2/M in 3T3 cells transduced with IKZF1 WT and KRK without induction (black) and 24h after induction with 4-OHT performed in triplicate. Pairwise t-tests were performed between IKZF1 WT and KRK, with the Bonferroni corrected p-adjusted value stated.
G. Missense mutations in the conserved helical motif region of *IKZF1, IKZF2,* and *IKZF3* detected in patients with hematopoietic malignancies, including pediatric B-ALL, T-ALL, Hairy cell leukemia, Hodgkin lymphoma, Diffuse large B-cell lymphoma, and Chronic myeloid leukemia. Five computational methods, Polyphen2, AlphaMissense, Fathmm, MutPred2, and Panther were used to predict the functional impact of each missense mutation. Prediction scores and references can be found in Supplementary Table 3.

Supplemental Methods

Chromatin-associated RNA-seq analysis

Sequencing reads were aligned against mm10 with nextflow nfcore/rnaseq:1.3 using star as aligner. Gene based read counts were obtained using featureCounts function from Rsubread Bioconductor package [1] and intronic reads were also included in quantification. Normalisation was performed in DESeq2 [2] and data was rlog transformed for PCA and heatmaps. Differential gene expression (DEG) analysis was conducted using DESeq2 with likelihood ratio test using parameter test= "LRT". DEGs were defined as those with Benjamini-Hochberg adjusted p-value (P) < 0.01 and a fold change >2 or <0.5 at any of the time points vs 0 hours. Heatmaps were generated using ComplexHeatmap [3]. K-means DEG clusters were generated with the parameter row_km = 5 with Heatmap function with set.seed(123) activated in R. Gene ontology analysis for each gene clusters were performed with goseq Bioconductor package (1.24; Ref: [4]).

ATAC-seq analysis

The reads were demultiplexed with bcl2fastq 2.20.0, allowing 0 mismatches. After demultiplexing, raw reads were processed with Nextflow nf-core/atacseq:1.0.0 (Ref: [5]) with parameters: '--genome GRCm38 -profile conda' using bwa (v0.7.17; Ref: [6]) aligner. ATACshift mode of deeptools (v. 2.3.5; Ref: [7]) alignmentSieve function was used to shift the reads with parameters: '--minFragmentLength 0 --maxFragmentLength 120' for Nucleosome Free Region (NFR – fragment length less than 120 bp). The filtered bam files were processed with deeptools alignment Sieve function with parameters: '--ATACshift --minFragmentLength 180' for Nucleosome Bound Region (NBR – fragment length more than 180 bp). Peaks were called wwith MACS2 (v2.1.2) callpeak function with parameters: '-f BAMPE -g mm' (Ref: [8]). Consensus peaks throughout different time points were defined by using nf-core/atacseq. Peaks overlapped with the black list mm10 v1 (Ref: [9],[10] were removed from downstream analysis. Peak-based read counts were then obtained using featureCounts (v1.6.4, Ref [1]). DESeq2 was used for differentially accessibility analyss (Ref: [2]). Peaks were annotated with ChIPseeker Bioconductor package[11].

BigWig files were generated based on BAM files from the NFR ATAC reads. Scale factors for each BAM file were calculated beforehand using the formula 1000000 divided by the number of mapped reads obtained from the Samtools (v.1.9; Ref: [12]) flagstat function. genomeCoverageBed function from Bedtools (v2.27.1; Ref:[13]) was applied with command: 'genomeCoverageBed -ibam $outDir/${SAMPLE}.mLb.ss.sorted.NFR.bam
-bg -scale $SCALE_FACTOR -pc | sort -k1,1 -k2,2n >
$outDir/bigwig_gcov/${SAMPLE}.mLb.ss.sorted.NFR.bedGraph'. Followed by deploying bedGraphToBigWig (Kent et al., 2010) with the following command: 'bedGraphToBigWig $outDir/bigwig_gcov/${SAMPLE}.mLb.ss.sorted.NFR.bedGraph $gsizes
$outDir/bigwig_gcov/${SAMPLE}.mLb.ss.sorted.NFR.bigWig'.

ChIP-seq analysis

Ikaros, H3K27ac, and CHD4 0, 1, and 2 hours Raw paired-end 41bp sequencing reads were aligned against mouse genome (mm10) using Bowtie2 (v. 2.4.2 [14]) with parameters: '--no-mixed --no-discordant'. Picard (v. 2.23.7; https://broadinstitute.github.io/picard/) SortSam and MarkDuplicates were applied for sorting and converting sam files to bam files and duplicated reads were marked. Peaks were called with MACS2 (v.2.2.7.1) callpeak function (with -f BAMPE -g mm for Ikaros and H3K27ac; with -f BAMPE --broad -g mm for CHD4). The consensus peaks across different time points were defined by using GenomicRanges [15] with the reduce function. Peak-based read counts were then obtained using the featureCounts function from Rsubread[1]. DESeq2 was used for differential H3K27ac analysis. For generation of Bigwig files, we used the 2h IP amount as the baseline to calculate the size factor (SF) for the downsampling analysis at each time point. The SF at each time point was calculated by dividing the IP amout at time 0 hours or 1 hour by the IP amout at 2 hours. Reads from raw fastq files were randomly selected based on the SF calculated for downsampling by using seqtk (v1.3; https://github.com/lh3/seqtk)

with 'seqtk sample -s100 $ori.fastq.gz $sizeFactor - | gzip - > $DS_fastq.gz'. After downsampling the fastq files, we applied the same analytical pipeline described in this section for alignment, sorting, converting to BAM files, and marking duplicated reads. We generated downsampled BigWig files using Deeptools (v. 3.5.0) with the following command: 'bamCoverage -b ChIP.DupMark.bam -p 16 -bl mm10-blacklist_v1.bed --extendReads --effectiveGenomeSize 2652783500 -o ChIP_MarkedDup.bw'.

For calibrated ChIP-seq samples KAP1 and H3K9me3, we analyzed this human-calibrated ChIP dataset based on the strategy by Fursova et al. 2019[16]. In summary, raw paired-end 41bp sequencing reads were aligned to a merged genome of mouse (mm10) and human (hg38) using Bowtie2 (v. 2.4.2), with prefixes Mm10 and Hg38 for mouse and human chromosome names, respectively. We employed Picard (v. 2.23.7) SortSam and MarkDuplicates to sort and convert the SAM file to BAM files, marking duplicated reads. The Samtools view function was applied to separate human and mouse-mapped reads using the following commands: samtools view -h -L $bedfile_hg38Chrs or samtools view -h -L $bedfile_mm10Chrs. We removed reads that mapped to both human and mouse and then separated the remaining reads based on whether they mapped to human or mouse. The downsampling factors (DFs) can be calculated as in Fursova et al., 2019[16]):

NGS Datasets

Bed files from published experiments were retrieved from GEO. Peaks from mm9 were converted to mm10 using UCSC LiftOver (https://genome.ucsc.edu/cgi-bin/hgLiftOver).

Dataset	genome	GEO accession	Reference	Source
Ctcf ChIP	mm10	GSE109671	Koohy et al., 2018[17]	Pre-B cells from young mice
Pre-B domains	mm10 lifted from mm9	GSE63525	Rao et al, 2014[18]	Pre-b lymphoblast CH12-LX

Super Enhancer Identification: H3K27ac peaks were utilized in the identification of super enhancers through the Rank Ordering of Super-Enhancers (ROSE; v.1.0.0) method (https://github.com/stjude/ROSE[19,20]) with parameters -s 12500 -t 0.

Enhancer Promoter connectivity analysis

Activation ratio + contact (AR+C) is a computational method for enhancer-promoter (E-P) assignment which represents a more recent implementation of the E-P activity correlation method described in Barešić et al.[21] 1) Activation ratio (AR) which captures how well the enhancer activity predicts the gene promoter activity and 2) Contact (C) - the frequency of E-P contact.

Activation Ratio was calculated using enhancer and promoter FANTOM5 CAGE data[22] across 395 mouse cell/tissue samples. The level of co-expression between putative E-P within 3Mb was quantified. 1) The enhancer was labelled as "inactive" if its expression was 0 TPM, and "active" otherwise. 2)The median expression of the candidate promoter was recorded for samples in which the enhancer was active (E+) and samples in which the enhancer was inactive (E-). Next, the activation ratio (AR) for that E-P pair was defined as the log-fold change in median promoter expression in E+ samples compared to E- samples. 3) The statistical significance of the observed activation ratio for that E-P pair was evaluated based on a permutation test. 4) Empirical P-values derived were then subjected to multiple testing correction. E-P pairs below the significance threshold are considered significantly co-expressed.

Contact: Enhancer-promoter pairs supported by 3D contact were identified using high-resolution Hi-C or Micro-C data from different cell types[23],[24],[25]. An individual E-P pair was marked as "supported by 3D contact" in each of the 4 cell types if its normalised contact frequency exceeded a predefined threshold representing the genome background contact frequency in that specific cell type (or contact matrix).

Combining AR + C: To obtain a single measure of enhancer responsiveness per gene, the median activation ratio across all its AR+C associations was estimated for each gene.

Permutation tests

Permutation tests were performed in R to determine whether Ikaros fast repressed genes are located closer to each other than expected based on the overall genomic distribution of genes. The number of co-occurrences of Fast repressed genes within 200kb windows was compared against 100,000 permutations in which the identity of fast repressed genes was randomised whilst gene positions remained invariant. The same approach was also taken to ask whether fast repressed genes tended to be located within the same pre-B contact domains.

Liquid chromatography-tandem mass spectrometry analysis

ChIP-MS. Chromatographic separation was performed using an Ultimate 3000 RSLC nano liquid chromatography system (Thermo Scientific) coupled to a Thermo Q-Exactive HFX mass spectrometer via an EASY-Spray source. Peptide solutions were injected and loaded onto a trapping column (Acclaim PepMap 100 C18, 100µm × 2cm) for desalting and concentration at 8µL/min in 2% acetonitrile, 0.1% TFA. Peptides were then eluted on-line to an analytical column (EASY-Spray PepMap RSLC C18, 75µm × 50cm) at a flow rate of 250nL/min. Peptides were separated using a 90 minute stepped gradient, 1-22% of buffer B for 60 minutes followed by 22-42% buffer B for another 30 minutes (composition of buffer A – 95/5%: H_2O/DMSO + 0.1% FA, buffer B – 75/20/5% MeCN/H_2O/DMSO + 0.1% FA) and subsequent column conditioning and equilibration. Eluted peptides were analysed by the mass spectrometer operating in positive polarity using a data-dependent acquisition mode. Ions for fragmentation were determined from an initial MS1 survey scan at 120,000 resolution, followed by HCD (Higher-energy Collision Induced Dissociation) of the top 30 most abundant ions at 15,000 resolution. MS1 and MS2 scan AGC targets were set to 3e6 and 5e4 for maximum injection times of 25ms and 50ms respectively. A survey scan m/z range of 350 – 1750 was used, with normalised collision energy set to 27%, charge state screening enabled with unassigned and +1 charge states rejected. A minimum AGC target was 8e3 with dynamic exclusion set to 50 seconds.

AP-MS. Chromatographic separation was performed using an Ultimate 3000 RSLC nano liquid chromatography (Thermo Scientific) coupled to an Orbitrap HFX mass spectrometer (Thermo Scientific) via an EASY-Spray source. Electro-spray nebulisation achieved by interfacing to Bruker PepSep emitters (PN: PSFSELJ20, 20µm). Peptide solutions were injected directly onto the analytical column (self-packed column, CSH C18 1.7µm beads, 300µm × 35cm) at working flow rate of 5µL/min for 8 minutes. Peptides were then separated using a 66 minute stepped gradient: 0-45% of buffer B for 66 minutes (composition of buffer A – 95/5%: H_2O/DMSO + 0.1% FA, buffer B – 75/20/5% MeCN/H_2O/DMSO + 0.1% FA), followed by column conditioning and equilibration. Eluted peptides were analysed by the mass spectrometer in positive polarity using a data-independent acquisition mode as follows: an initial MS1 scan was carried out at 120,000 resolution with an AGC target of 3e6 for a maximum IT of 200ms, m/z range: 350-1650. This was followed by sequential MS2 acquisition and fragmentation of ions at 30,000 resolution over 26 variable windows. AGC target set to 3e6 with maximum IT on auto. Normalised collision energy was set to 27%.

Mass spectrometry raw data processing

ChIP-MS. Data were processed using the MaxQuant software platform (v1.6.10.43[26], with database searches carried out by the in-built Andromeda search engine against the Swissprot mus musculus database (downloaded – 20230504, entries – 21,965). A reverse decoy database approach was used at a 1% false discovery rate (FDR) for peptide spectrum matches. Search parameters were as follows:

maximum missed cleavages set to 2, fixed modification of cysteine carbamidomethylation and variable modifications of methionine oxidation, protein N-terminal acetylation, asparagine deamidation and cyclisation of glutamine to pyro-glutamate. Label-free quantification was enabled with an LFQ minimum ratio count of 1. 'Match between runs' function was used with match and alignment time limits of 0.7 and 20 minutes respectively.

AP-MS. Data were processed using the Spectronaut (Biognosys, v18.5.231110.55695) [27]. Analysis was performed in direct DIA mode: 1. ***Pulsar Search***: library generation and database search using default settings for a tryspin/p specific digest as follows - missed cleavage rate 3 and variable modifications allowed for methionine oxidation, protein N-terminal acetylation, asparagine deamidation and cyclisation of glutamine to pyro-glutamate. PSM, Peptide and Protein group FDR = 0.01. Searches were carried out against the Swissprot mus musculus protein sequence database concatenated with truncated sequence of IKZF1_MOUSE (Uniprot ID: Q03267, downloaded 31/012023, 21,967 entries). Truncated sequence determined by Clustal Omega multi-sequence alignment of canonical sequence and IKZF1 variants. Longest stretch of common sequence, concatenated into database as IKZF1 entry. We reasoned that this would help determine more accurately the relative level of IZF1 across conditions, as quantification would be determined from shared tryptic peptides only. Additionally, searches were also carried against a universal protein contaminants database[28] (downloaded 20220604, 381 entries). 2. ***Direct DIA analysis***: a mutated decoy database approach was employed with protein q-value cut-off for the experiment set to 0.01 at the identification level. Quantification set to MS2 with proteotypicity filter set to only protein group specific with no value imputation strategy employed. Protein quantification method set to MaxLFQ[29].

Chromatin-associated RNA-seq Buffers

Lysis Buffer	10 mM Tris pH 7.5, 10 mM KCl, 1.5 mM $MgCl_2$, 0.1% NP-40
Urea Buffer	10 mM Hepes pH 7.6, 150 mM NaCl, 3 mM $MgCl_2$, 0.5% NP-40, 0.1 mM EDTA, 10% glycerol, 0.5M Urea (freshly made)

Atac-seq Buffers

Lysis Buffer	10 mM Tris pH 7.5, 10 mM NaCl, 3 mM $MgCl_2$, 0.1% NP-40, 0.1% Tween-20, 0.01% Digitonin
5xTagmentation Buffer	50 mM TAPS, 25 mM $MgCl_2$, 50% dimethylformamide

ChIP-seq and ChIP-MS Buffers

Lysis Buffer	50 mM Hepes pH 7.5, 140 mM NaCl, 1 mM EDTA, 10% glycerol, 0.5% NP-40, 0.25% Triton
Nuclei Lysis Buffer	10 mM Tris pH 8, 100 mM NaCl, 1 mM EDTA, 0.5 mM EGTA, 0.1% sodium deoxycholate, 0.5% N-lauroyl sarcosine
Dilution Buffer	20 mM Tris pH 8, 150 mM NaCl, 1 mM EDTA, 1% Triton X-100
Low Salt Buffer	20 mM Tris pH 8, 150 mM NaCl, 2 mM EDTA, 1% Triton X-100, 0.1% SDS
High Salt Buffer	20 mM Tris pH 8, 500 mM NaCl, 2 mM EDTA, 1% Triton X-100, 0.1% SDS
LiCl Buffer	10 mM Tris pH 8, 250 mM LiCl, 1 mM EDTA, 1% NP-40, 1% sodium deoxycholate
TE	10 mM Tris pH 8, 1mM EDTA
ChIP Elution Buffer	100 mM $NaHCO_3$, 1% SDS

Affinity Purification MS Buffers

Lysis Buffer	10 mM Tris pH 7.5, 10 mM KCl, 1.5 mM $MgCl_2$, 0.1% NP-40, 0.1% Tween-20
Nuclear Extraction Buffer	10 mM Hepes pH 7.5, 300 mM NaCl, 5% glycerol, 1.5 mM $MgCl_2$, 0.2 mM EDTA
Wash Buffer	20 mM Tris pH 8, 150 mM NaCl, 0.2% NP-40, 5% glycerol

RT-qPCR Primers

Primer (mouse)	Forward	Reverse
Ccnd2 (nascent transcript)	AGTCCTAAGGCTCAGACGGT	CAGAGGGTGTTGCGACCTAA
Igll1 (nascent transcript)	ACACCCATGGCCTCAATAGC	AAGGTACGCGTGTAGGTCCA
Plekho2 (nascent transcript)	AGCCCAGTGTCACAAAACCA	TCCCTGACTCTAGACTGCCC
Endod1 (nascent transcript)	ATGTTGCTGAGGGGATGTGG	GAACTGGGAGACAGCCCTTG
Bcl11a (nascent transcript)	TGCCCCTGAAGCAAAGTTCT	ACTGTTGGGAATCGTGGGAG
Ubc (Housekeeping)	AGGAGGCTGATGAAGGAGCTTGA	TGGTTTGAATGGATACTCTGCTGGA
Ywhaz (Housekeeping)	CGTTGTAGGAGCCCGTAGGTCAT	TCTGGTTGCGAAGCATTGGG

ChIP-qPCR Primers

Primer (mouse)	Forward	Reverse
Ccnd2 Promoter	CCTTCCCTCCGAACTGCC	ATCTAACTGCCCTTCCAGCTT
Ccnd2 Enhancer	GAGGGAGGAAGTGAAGGCTG	CAGGAGGACAACAAACCCCA
Endod1 Enhancer	TGGATGGAGGAGGGACAGAG	AGGGAGTTAGGCAGGCTCAT
Gfra2 Enhancer	ACCTCCACTCAGGACAGAGG	ACAAGGGCTTTTGGACACGA
Plekho2 Enhancer	CCAGACGCTTTGAGTTGCTC	TCCTAGGTTCGTAGGCCACT
Lgals9 Enhancer	CTCCGCGTCACTTTCCTTCT	AGAACTGGGAGCTGTCCTCT
Bcl11a Enhancer	GGCAACTTACAGGATGCCCT	CACAACTTTCTTGGGCCTGC
Igll1 Promoter	CAAACCCCAGGCTGTCTCTA	GGCAGCTGTGAGTGAAAACA
Cdx2 Promoter (negative control)	ACCACCTTCTGCCTGAGAATGTAC	CCTCCAATCACAGGTTCAAAGACT
Gene desert (negative control)	TGCATGAGCAGAGGACTAGG	AGAAGTGCAAGCTCAGAACCTT

Antibodies

Antibody	Catalog no.	Usage
HA	Enzo ENZ-ABS120	WB: 1:1000 IF: 1: 400
HA Magnetic Beads	Thermo Scientific Pierce 88836	ChIP: 25µL per IP
H3	Abcam ab1791	WB: 1:1000
RBBP4	Abcam ab79416	WB: 1:1000
H3K27ac	Active Motif 39133	ChIP: 5µg per IP
H3K9me3	Diagenode C15410193	ChIP: 5µg per IP
KAP1	Abcam ab10483	ChIP: 5µg per IP
CHD4	Abcam ab70469	ChIP: 5µg per IP
IKZF1 (detects WT and DN Ik6 isoform)	Cell Signalling Technology	WB: 1:1000
MBD3	Abcam ab157464	WB: 1:1000
HDAC1	Abcam ab7028	WB: 1:1000

Data Availability

Proteomics data generated for this study have been deposited in PRIDE PXD050989 and PXD050986 and NGS data in GEO under accession number GSE256299.

Supplemental References

1 Liao, Y., Smyth, G. K. & Shi, W. The R package Rsubread is easier, faster, cheaper and better for alignment and quantification of RNA sequencing reads. *Nucleic Acids Res* **47**, e47, doi:10.1093/nar/gkz114 (2019).

2 Love, M. I., Huber, W. & Anders, S. Moderated estimation of fold change and dispersion for RNA-seq data with DESeq2. *Genome Biol* **15**, 550, doi:10.1186/s13059-014-0550-8 (2014).

3 Gu, Z., Eils, R. & Schlesner, M. Complex heatmaps reveal patterns and correlations in multidimensional genomic data. *Bioinformatics* **32**, 2847-2849, doi:10.1093/bioinformatics/btw313 (2016).

4 Young, M. D., Wakefield, M. J., Smyth, G. K. & Oshlack, A. Gene ontology analysis for RNA-seq: accounting for selection bias. *Genome Biol* **11**, R14, doi:10.1186/gb-2010-11-2-r14 (2010).

5 Ewels, P. A. *et al.* The nf-core framework for community-curated bioinformatics pipelines. *Nat Biotechnol* **38**, 276-278, doi:10.1038/s41587-020-0439-x (2020).

6 Li, H. & Durbin, R. Fast and accurate short read alignment with Burrows-Wheeler transform. *Bioinformatics* **25**, 1754-1760, doi:10.1093/bioinformatics/btp324 (2009).

7 Ramirez, F. *et al.* deepTools2: a next generation web server for deep-sequencing data analysis. *Nucleic Acids Res* **44**, W160-165, doi:10.1093/nar/gkw257 (2016).

8 Zhang, Y. *et al.* Model-based analysis of ChIP-Seq (MACS). *Genome Biol* **9**, R137, doi:10.1186/gb-2008-9-9-r137 (2008).

9 Amemiya, H. M., Kundaje, A. & Boyle, A. P. The ENCODE Blacklist: Identification of Problematic Regions of the Genome. *Sci Rep* **9**, 9354, doi:10.1038/s41598-019-45839-z (2019).

10 Consortium, E. P. An integrated encyclopedia of DNA elements in the human genome. *Nature* **489**, 57-74, doi:10.1038/nature11247 (2012).

11 Yu, G., Wang, L. G. & He, Q. Y. ChIPseeker: an R/Bioconductor package for ChIP peak annotation, comparison and visualization. *Bioinformatics* **31**, 2382-2383, doi:10.1093/bioinformatics/btv145 (2015).

12 Danecek, P. *et al.* Twelve years of SAMtools and BCFtools. *Gigascience* **10**, doi:10.1093/gigascience/giab008 (2021).

13 Quinlan, A. R. & Hall, I. M. BEDTools: a flexible suite of utilities for comparing genomic features. *Bioinformatics* **26**, 841-842, doi:10.1093/bioinformatics/btq033 (2010).

14 Langmead, B. & Salzberg, S. L. Fast gapped-read alignment with Bowtie 2. *Nat Methods* **9**, 357-359, doi:10.1038/nmeth.1923 (2012).

15 Lawrence, M. *et al.* Software for computing and annotating genomic ranges. *PLoS Comput Biol* **9**, e1003118, doi:10.1371/journal.pcbi.1003118 (2013).

16 Fursova, N. A. *et al.* Synergy between Variant PRC1 Complexes Defines Polycomb-Mediated Gene Repression. *Mol Cell* **74**, 1020-1036 e1028, doi:10.1016/j.molcel.2019.03.024 (2019).

17 Koohy, H. *et al.* Genome organization and chromatin analysis identify transcriptional downregulation of insulin-like growth factor signaling as a hallmark of aging in developing B cells. *Genome Biol* **19**, 126, doi:10.1186/s13059-018-1489-y (2018).

18 Rao, S. S. *et al.* A 3D map of the human genome at kilobase resolution reveals principles of chromatin looping. *Cell* **159**, 1665-1680, doi:10.1016/j.cell.2014.11.021 (2014).

19 Loven, J. *et al.* Selective inhibition of tumor oncogenes by disruption of super-enhancers. *Cell* **153**, 320-334, doi:10.1016/j.cell.2013.03.036 (2013).
20 Whyte, W. A. *et al.* Master transcription factors and mediator establish super-enhancers at key cell identity genes. *Cell* **153**, 307-319, doi:10.1016/j.cell.2013.03.035 (2013).
21 Baresic, A., Nash, A. J., Dahoun, T., Howes, O. & Lenhard, B. Understanding the genetics of neuropsychiatric disorders: the potential role of genomic regulatory blocks. *Mol Psychiatry* **25**, 6-18, doi:10.1038/s41380-019-0518-x (2020).
22 Consortium, F. *et al.* A promoter-level mammalian expression atlas. *Nature* **507**, 462-470, doi:10.1038/nature13182 (2014).
23 Bonev, B. *et al.* Multiscale 3D Genome Rewiring during Mouse Neural Development. *Cell* **171**, 557-572 e524, doi:10.1016/j.cell.2017.09.043 (2017).
24 Hill, L. *et al.* Igh and Igk loci use different folding principles for V gene recombination due to distinct chromosomal architectures of pro-B and pre-B cells. *Nat Commun* **14**, 2316, doi:10.1038/s41467-023-37994-9 (2023).
25 Hsieh, T. S. *et al.* Resolving the 3D Landscape of Transcription-Linked Mammalian Chromatin Folding. *Mol Cell* **78**, 539-553 e538, doi:10.1016/j.molcel.2020.03.002 (2020).
26 Tyanova, S., Temu, T. & Cox, J. The MaxQuant computational platform for mass spectrometry-based shotgun proteomics. *Nat Protoc* **11**, 2301-2319, doi:10.1038/nprot.2016.136 (2016).
27 Bruderer, R. *et al.* Extending the limits of quantitative proteome profiling with data-independent acquisition and application to acetaminophen-treated three-dimensional liver microtissues. *Mol Cell Proteomics* **14**, 1400-1410, doi:10.1074/mcp.M114.044305 (2015).
28 Turriziani, B. *et al.* On-beads digestion in conjunction with data-dependent mass spectrometry: a shortcut to quantitative and dynamic interaction proteomics. *Biology (Basel)* **3**, 320-332, doi:10.3390/biology3020320 (2014).
29 Cox, J. *et al.* Accurate proteome-wide label-free quantification by delayed normalization and maximal peptide ratio extraction, termed MaxLFQ. *Mol Cell Proteomics* **13**, 2513-2526, doi:10.1074/mcp.M113.031591 (2014).
